# A maximum-entropy length-orientation closure for short-fiber reinforced composites

**DOI:** 10.1007/s00466-024-02447-7

**Published:** 2024-02-24

**Authors:** Alok Mehta, Matti Schneider

**Affiliations:** 1 Institute of Engineering Mathematics, University of Duisburg-Essen, Essen, Germany; 2 Fraunhofer Institute for Industrial Mathematics ITWM, Kaiserslautern, Germany

**Keywords:** Short-fiber composite, Representative volume element, Length-orientation distribution coupling, Maximum entropy closure, Sequential addition and migration

## Abstract

We describe an algorithm for generating fiber-filled volume elements for use in computational homogenization schemes which accounts for a coupling of the fiber-length and the fiber-orientation. For prescribed fiber-length distribution and fiber-orientation tensor of second order, a maximum-entropy estimate is used to produce a fiber-length-orientation distribution which mimics real injection molded specimens, where longer fibers show a stronger alignment than shorter fibers. We derive the length-orientation closure from scratch, discuss its integration into the sequential addition and migration algorithm for generating fiber-filled microstructures for industrial volume fractions and investigate the resulting effective elastic properties. We demonstrate that accounting for the length-orientation coupling permits to match the measured Young’s moduli in principal fiber direction and transverse to it more accurately than for closure approximations ignoring the length-orientation coupling.

## Introduction

### State of the art

Lightweight components made of short-fiber reinforced plastics and manufactured by injection molding combine advantageous mechanical properties, a high design freedom and short cycle times [1]. Due to the cylindrical reinforcements, the effective mechanical properties of such a composite material are anisotropic, in general, and depend on the fiber characteristics, i.e., the fiber-volume fraction and the realized orientation state [2–4]. Mechanically characterizing such a material system may involve significant effort, essentially due to the anisotropy of the material and the various attained fiber-orientation states, in particular when long-term experiments like creep or fatigue are involved [5–7].

To alleviate these costs, it may be beneficial to rely on computational approaches complementing a few basic mechanical experiments. Quite a number of such computational multiscale methods have been developed, and we refer to the pertinent review articles [9–11] for an overview of the ideas underlying these developments.

These micromechanics methods consider the microstructure of the composite at the starting point, so that the mechanical behavior of the material will emerge from this microstructural construction plan if the proper mechanical models for the fiber and the matrix (and, possibly, the interface) are supplemented. The theoretical foundation for these approaches is provided by the mathematical theory of *homogenization* [12, 13].

**Fig. 1:**
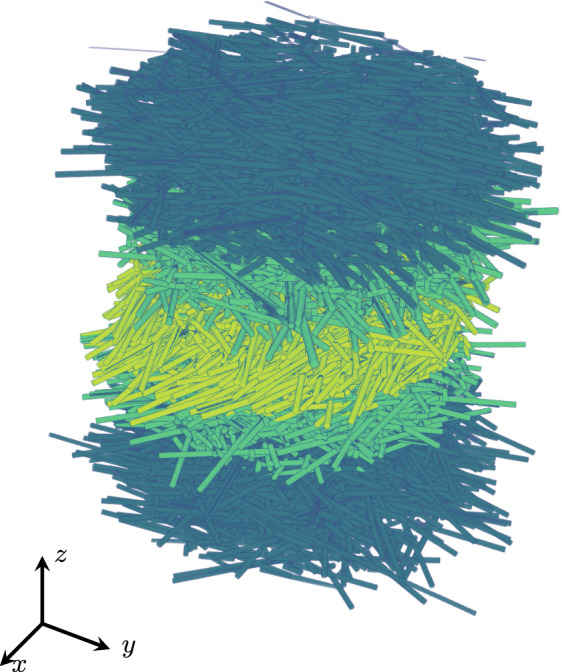
3D $\upmu$-CT image of an SFRP [8]

In case of fiber composites, the considered microstructures are actually random as a result of the manufacturing process. Moreover, the microstructures often comes with a high degree of complexity, see Fig. 1. Fortunately, modern digital image processing methods like micro-computed tomography [14–16] ($\upmu$CT) offer detailed insights into these complex microstructures.

Returning to the engineering design process of the desired injection-molded components, it is imperative to have accurate simulation tools of the injection-molding process at hand. This process involves filling a cavity with a polymer melt in which the fibers are suspended. Apart from the obviously complex physics underlying a proper modeling approach of injection molding there is another challenge concerning what we would nowadays call data management. Indeed, the fiber-orientation state needs to be described at every continuum point in space and time during the flow. Advani and Tucker [17] proposed to use fiber-orientation tensors, a compact *tensorial* characteristic of the fiber-orientation state, as such a descriptor and useful basis of injection-molding simulation. To this day, second-order fiber-orientation tensors are most widely used for such simulations [18, 19]. In particular, they represent standard for both industrial and academic injection-molding simulations. However, both for modeling the flow and for estimating the effective mechanical properties, fiber-orientation tensors of higher order, possessing additional information, are required. Clearly, this additional information cannot be recovered from the second-order tensors. However, Folgar and Tucker [20] proposed to use so-called *closure approximations*, i.e., relations which express the sought fiber-orientation tensors of higher order as functions of the available lower-order fiber-orientation tensor. This strategy bypassed the lack of additional information by providing “plausible” higher-order fiber-orientation tensors for given lower-order tensors based on what may be called “expert’s knowledge”. Quite a number of these closure approximations were introduced [21–26], leading to a robust simulation technology which is critical for lightweight design.

The potential gains of multiscale modeling strategies will be particularly high if the mechanical characterization is rather expensive, e.g., when long time scales are involved and geometric anisotropy is present. For instance, fatigue experiments may take up to several weeks or even months to complete [5, 6]. The accompanying multiscale techniques need to account for the material degradation, and *full-field* computational modeling approaches are necessary to resolve fine details of this degradation like cracks emerging at fiber tips and growing steadily [27–29]. For such approaches, it is necessary to provide suitable computational cells serving as the geometry to work on.

More often than not relying upon real digital images for these geometries is not recommended. Indeed, apart from the involved expenses there are a few disadvantages of real images. For a start, these images typically involve artifacts. Secondly, it is difficult to cover all the fiber-volume and fiber-orientation states of interest with sufficient accuracy. Last but not least, real digital images are non-periodic by construction. In contrast, it is well-known that working with periodic microstructures and periodic boundary conditions permits to drastically reduce the computational effort of multiscale methods [30–32].

These reasons motivate studying microstructure modeling and generation tools [33, 34]. For short-fiber composites, simplistic approaches like random sequential adsorption [35–37] fail to produce the high volume fractions used in industry for fiber-orientation states that are not well-aligned. Therefore, a number of alternative, more sophisticated approaches needed to be considered, e.g., based on full finite-element models and an explicit compression simulation [38], random-walk based models of curved fibers [39] or the shaking and breaking approach of Li et al. [40].

Based on work for isotropic composites with spherocylindrical fibers [41], the Sequential Addition and Migration (SAM) method [42] encodes the microstructure-generation problem of short non-overlapping cylindrical fibers with prescribed fiber-orientation tensor of fourth order as an optimization problem and uses a gradient-descent method to solve the problem. With some insights from computer science, the method was demonstrated to produce short-fiber microstructures with high fidelity and industrial filler fraction in a robust manner [43, 44]. Subsequent extensions of the method concerned accounting for a fiber-length distribution [45] and non-straight, i.e., curved fibers [46].

### Contributions

Modern closure approximations [25, 26] provide a full fiber-orientation distribution (estimate) for a given second-order fiber-orientation tensor. Moreover, fiber-length data is often available, e.g., from incineration [47, 48] or from segmented $\upmu$CT scans.

Suppose the results of an injection-molding simulation are available, together with a fiber-length distribution (either discrete or continuous). Then, to conduct mechanical multiscale simulations [7, 49, 50], it is required to generate appropriate volume elements matching the desired composite characteristics such as the fiber-volume fraction, the fiber-length distribution and the fiber-orientation distribution. The standard approach [40, 45, 51] is to draw that fiber length and the fiber direction *independently*, i.e., to assume that the fiber length and the fiber direction are not correlated. Being even more explicit this assumption means that if we restrict our attention to very short fibers or to very long fibers, the emerging fiber-orientation distributions will be identical! However, this is not what we observe in real short-fiber composites.

Let us take a look at $\upmu$CT data from Müller’s thesis [8], more precisely the microstructure shown in Fig. 1 and comprising a glass-fiber reinforced PBT, see Sect. 4 for further details. The structure shows different layers in which fibers are aligned differently. On top and on the bottom, the “skin” layers are shown which emerge from the polymer flow close to the wall. In the center, the so-called “core” layer is shown, where the fiber orientation is typically completely different from the skin layers [18, 19]. Moreover, we highlighted two further “transitional” layers.

**Fig. 2:**
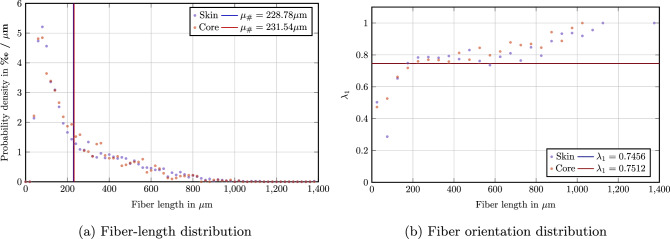
Length and length-orientation distribution in two different layers of the specimen shown in Fig. 1

Figure 2 provides information about both the fiber-length and the fiber-orientation distribution. Figure 2a shows the fiber-length distribution present in the skin and the core layer. We observe that the length distributions of both layers turns out to be rather similar with a (number-weighted) mean fiber length $\mu _{\#}$ of about $230\,\upmu$m.

Figure 2b shows the largest eigenvalue $\lambda _1$ of the second-order fiber-orientation tensor as a function of the fiber length for both layers. Keep in mind that the fiber orientations of both layers are rather unlike, i.e., the fibers tend to align in flow direction in the skin layer, whereas an alignment transverse to the flow is observed in the core layer. However, due to the eigenvalue analysis, these orientational issues are factored out, and only the magnitude of fiber orientation in the principal direction is shown.

In either case, Fig. 2b reveals a distinct coupling of the fiber orientation and the fiber length. Indeed, if such a coupling was not present, the fiber orientation ought to be a constant function of the fiber length. In contrast, Fig. 2b reveals shorter fibers to be more isotropic, i.e., with principal fiber orientations $\lambda _1$ closer to 1/3 (corresponding to the isotropic or cubic fiber-orientation state) and longer fibers tending to be more aligned with $\lambda _1 \approx 1$. Moreover, we observe that the principal fiber orientation is increasing with the fiber length.

Incidentally, Fig. 2b reveals even more, namely that the fiber length-orientation coupling in both the skin and the core layer is very much alike. Thus, it appears reasonable to design a model for the length-orientation function based on the fiber-orientation state and the length distribution.

These experimental observations motivated the article at hand. The purpose of this paper is to provide a closure approximation of the full fiber length-orientation distribution function based on the second-order fiber-orientation tensor and a prescribed fiber-length distribution, see Sect. 2. We will provide this approximation based on the maximum-entropy estimate (MEE) which is well-studied in a different context [52–54]. We discuss the integration of the model into the SAM framework, see Sect. 3, and discuss the implications for the emerging effective elastic properties, see Sect. 4.

## Describing short-fiber microstructures

### Fiber-orientation and fiber-length distributions

We consider short-fiber reinforced composites, i.e., we assume that each fiber in such a composite may be described by a straight cylinder with length $\ell$, principal axis ${\varvec{p}}$ and diameter *D*. Typically, the variations of the diameter between different fibers of a composite is negligible, whereas both the fiber length $\ell$ and the fiber orientation ${\varvec{p}}$ vary significantly. The latter two characteristics may be described in terms of a length-orientation distribution function

2.1 $$\begin{aligned} f : \mathbb {R}_{>0} \times S^2 \rightarrow \mathbb {R}_{\ge 0}, \quad (\ell ,{\varvec{p}}) \mapsto f (\ell ,{\varvec{p}}), \end{aligned}$$

where $S^2$ denotes the unit sphere in $\mathbb {R}^3$. The length-orientation distribution function is a probability distribution, i.e., non-negative and integrates to unity, which satisfies the symmetry condition

2.2 $$\begin{aligned} f (\ell ,{\varvec{p}}) = f (\ell ,-{\varvec{p}}) \quad \text {for all} \quad \ell >0 \quad \text {and} \quad {\varvec{p}}\in S^2, \end{aligned}$$

which reflects the sign ambiguity when describing a cylinder in terms of its principal axis.

In practice, the full length-orientation distribution function $$f $$ is not known, and only partial information and estimates are available. A classical way to estimate the fiber-length distribution $$\psi  :\mathbb {R}_{>0} \rightarrow \mathbb {R}_{\ge 0}$$, which may be recovered from the length-orientation distribution (2.1) by integrating over orientation space

2.3 $$\begin{aligned} \psi  (\ell ) = \displaystyle \int _{S^2} {f (\ell ,{\varvec{p}})} \, dS, \quad \ell >0, \end{aligned}$$

proceeds via incineration of the matrix material (see, e.g., Table 1 in Goris et al. [55]), and counting the fiber length of the individual fibers under the microscope. In particular, the connection between fiber length and orientation is lost.

Fiber-orientation data is typically encoded via fiber-orientation tensors [17, 56], which can be determined from $$\upmu $$CT images [39, 57–60]. Fiber-orientation tensors correspond to moments of the length-orientation distribution $$f $$, and the two most popular fiber-orientation tensors are of second

2.4 $$\begin{aligned} {\varvec{A}} = \frac{1}{\bar{\ell }} \displaystyle \int _0^\infty \!\!\int _{S^2} { \ell \, {\varvec{p}}\otimes {\varvec{p}}\, f {(\ell ,{\varvec{p}})}} \, dS \,d\ell \end{aligned}$$

and fourth order,

2.5 $$\begin{aligned} \mathbb {A}  = \frac{1}{\bar{\ell }} \displaystyle \int _0^\infty \!\!\int _{S^2} { \ell \, {\varvec{p}}\otimes {\varvec{p}}\otimes {\varvec{p}}\otimes {\varvec{p}}\, f {(\ell ,{\varvec{p}})}.} \, dS \,d\ell \end{aligned}$$

These tensors are normalized by the average fiber length

2.6 $$\begin{aligned} \bar{\ell } = \displaystyle \int _0^\infty {\ell \psi  {(\ell )}} \, d\ell \end{aligned}$$

to ensure the normalization conditions

2.7 $$\begin{aligned} \mathbb {A} :{\varvec{I}}= {\varvec{A}}  \quad \text {and} \quad {\varvec{A}} :{\varvec{I}}= 1 \end{aligned}$$

to hold in terms of the second-order identity tensor ${\varvec{I}}$ and the double contraction which is denoted by a colon. Fiber-orientation tensors have their roots in injection-molding simulations where the second-order fiber-orientation tensor ${\varvec{A}}$ is often the only information available [18, 20]. Although considering higher moments in the length variable $\ell$ is conceivable, the length-averaged forms (2.4) and (2.5) are the most natural, as they arise from a volume averaging of the cylinders. In particular, this form typically arises when computing fiber-orientation tensors from $\upmu$CT images [61].

Classically, for short-fiber reinforced composites, the details of the fiber-length distribution are ignored, and only the mean fiber length $\bar{\ell }$ is considered. Put differently, this means that the fiber-length distribution $\psi$ is assumed to be concentrated at the specific fiber length $\bar{\ell }$. In this case, the fiber-orientation distribution

2.8 $$\begin{aligned} \varphi  :S^2 \rightarrow \mathbb {R}_{\ge 0}, \quad {\varvec{p}}\mapsto \varphi  ({\varvec{p}}), \end{aligned}$$

carries all relevant information about the length-orientation characteristics of the composite.

Apparently, there is a gap between the data which is available and the data which would be necessary for estimating the effective mechanical properties of short-fiber composites. For this purpose, a number of so-called closure approximations were developed, see Kugler et al. [25] for a recent review. In the past, closure approximations were considered as approximations of higher-order fiber-orientation tensors in terms of lower-order fiber-orientation terms. However, this point of view permitted certain pathologies, which lead to unphysical or mathematically contradictory properties of the estimated higher-order tensors. For instance, the quadratic, the linear and the hybrid closure, introduced by Advani and Tucker [17], do not arise from a fiber-orientation distribution, in general.

Over time, it became apparent that it is more convenient to consider closure approximations which provide an estimate of the entire fiber-orientation distribution function $\varphi$ based on prescribed fiber-orientation tensors. The two most popular closures of this type, taking the second-order fiber-orientation tensor ${\varvec{A}}$ as input are the exact closure [23, 24, 62], which is based on the fiber-orientation distribution function^1^

2.9 $$\begin{aligned} \varphi ^\texttt {ACG}_{{\varvec{M}}}({\varvec{p}}) = \frac{1}{4\pi } \, \left( {\varvec{p}}^T {\varvec{M}}{\varvec{p}}\right) ^{-\frac{3}{2}}, \quad {\varvec{p}}\in S^2, \end{aligned}$$

in terms of a symmetric, positive definite and unimodular^2^$3 \times 3$-matrix ${\varvec{M}}$, and the maximum entropy closure^3^ [3, 22]

2.10 $$\begin{aligned} \varphi ^\texttt {Bingham}_{{\varvec{M}}}({\varvec{p}}) = \exp \left( {\varvec{p}}^T {\varvec{M}}{\varvec{p}}- c({\varvec{M}}) \right) , \quad {\varvec{p}}\in S^2, \end{aligned}$$

with a symmetric $3 \times 3$-matrix ${\varvec{M}}$ and the normalization constant

2.11 $$\begin{aligned} c({\varvec{M}}) = \log \displaystyle \int _{S^2} { \exp \left( {\varvec{p}}^T {\varvec{M}}{\varvec{p}}\right) } \, dS. \end{aligned}$$

The exact closure is based on an exact solution of the fiber-orientation dynamics (with vanishing Folgar–Tucker diffusivity [20]), see Montgomery-Smith et al. [23, 24], whereas the maximum entropy closure [3, 22] maximizes the information-theoretic entropy. In either of the two cases, for prescribed second-order fiber-orientation tensor ${\varvec{A}}$, the matrix ${\varvec{M}}$ may be determined (numerically) as a solution of the equation

2.12 $$\begin{aligned} \displaystyle \int _{S^2} { {\varvec{p}}\otimes {\varvec{p}}\, \varphi  } \, dS{\mathop {=}\limits ^{!}} {\varvec{A}} . \end{aligned}$$

Some care has to be taken if the fiber-orientation tensor ${\varvec{A}}$ is singular or close to singular, i.e., $\det {\varvec{A}}  \approx 0$ holds. In any case, with the estimated fiber-orientation distribution $\varphi$ at hand, the necessary higher-order information (like the fourth-order fiber-orientation tensor $\mathbb {A}$) can be extracted. Of course, lost information cannot be recovered this way.

### A maximum-entropy length-orientation closure

We consider the following problem. Suppose that only the fiber-length distribution $$\psi  $$ and the second-order fiber-orientation tensor $${\varvec{A}} $$ are known. Then, we wish to infer a suitable estimate for the length-orientation distribution function $$f $$ which is compatible in the sense that Eqs. (2.3) and (2.4) hold, i.e., the conditions

2.13 $$\begin{aligned}  &   \displaystyle \int _0^\infty \!\!\int _{S^2} { \ell \, {\varvec{p}}\otimes {\varvec{p}}\, f (\ell ,{\varvec{p}})} \, dS \,d\ell = \bar{\ell }\,{\varvec{A}}  \quad \text {and} \nonumber \\  &   \displaystyle \int _{S^2} {f (\ell ,{\varvec{p}})} \, dS = \psi  (\ell ), \quad \ell >0, \end{aligned}$$

are satisfied involving the mean length $\bar{\ell }$ (2.6). A pragmatic way of ensuring these conditions to hold proceeds by assuming that the fiber length and fiber orientation are not coupled, i.e., the functional form

2.14 $$\begin{aligned} f (\ell ,{\varvec{p}}) = \psi  (\ell ) \varphi  ({\varvec{p}}), \quad \ell >0, \quad {\varvec{p}}\in S^2, \end{aligned}$$

is postulated. The relationship (2.14) means that, for each two fiber lengths $\ell$ and $\ell '$, the orientation distributions of fibers of length $\ell$ and $\ell '$, respectively, are completely identical. Experimental evidence, see Wang et al. [65] and Müller [8], suggests that this is not true, as shorter fibers are subject to fewer geometrical restrictions compared to longer fibers and thus tend to orient much faster during the flow, see also Sect. 1. Still, the uncoupled model (2.14) is a simple and straightforward approach, and permits using previously established technology, e.g., the exact and maximum entropy closure, see Eqs. (2.9) and (2.10).

As an alternative, we will consider the maximum entropy closure in the context of the length-orientation distribution, i.e., we seek a maximizer of the information-theoretic entropy functional

2.15 $$\begin{aligned}  &   H(f ) \longrightarrow \max _{\text {such that }(2.13)} \quad \text {with} \quad \nonumber \\  &   H(f )= - \displaystyle \int _0^\infty \!\!\int _{S^2} { f (\ell ,{\varvec{p}}) \log f(\ell ,{\varvec{p}}) - f(\ell ,{\varvec{p}})} \, dS \,d\ell .\nonumber \\ \end{aligned}$$

To solve this optimization problem, we enforce the constraints in terms of suitable Lagrange multipliers, a traceless, symmetric $3 \times 3$-matrix ${\varvec{B}}\in \text {Sym}_0(3)$ and a function $\zeta :\mathbb {R}_{\ge 0} \rightarrow \mathbb {R}$, and consider the Lagrangian function

2.16 $$\begin{aligned} L(f,{\varvec{B}},\zeta )= &   - \displaystyle \int _0^\infty \!\!\int _{S^2} { f (\ell ,{\varvec{p}}) \log f(\ell ,{\varvec{p}}) - f(\ell ,{\varvec{p}})} \, dS \,d\ell \nonumber \\  &   + {\varvec{B}}:\left( \displaystyle \int _0^\infty \!\!\int _{S^2} { \ell \, {\varvec{p}}\otimes {\varvec{p}}\, f (\ell ,{\varvec{p}})} \, dS \,d\ell - \bar{\ell }\,{\varvec{A}}  \right) \nonumber \\  &   + \displaystyle \int _0^\infty { \zeta (\ell ) \left( \displaystyle \int _{S^2} {f (\ell ,{\varvec{p}})} \, dS - \psi  (\ell ) \right) } \, d\ell .\nonumber \\ \end{aligned}$$

Rearranging the terms in the expression of the Lagrangian,

2.17 $$\begin{aligned} L(f,{\varvec{B}},\zeta )= &   \displaystyle \int _0^\infty \!\!\int _{S^2} {-} \, dS \,d\ell f (\ell ,{\varvec{p}}) \log f(\ell ,{\varvec{p}}) + f(\ell ,{\varvec{p}}) \nonumber \\  &   + \,\ell \, {\varvec{B}}: {\varvec{p}}\otimes {\varvec{p}}\, f (\ell ,{\varvec{p}}) + \zeta (\ell ) f (\ell ,{\varvec{p}}) \nonumber \\  &   - \,\bar{\ell }\,{\varvec{B}}:{\varvec{A}}  - \displaystyle \int _0^\infty {\zeta (\ell ) \psi  (\ell )} \, d\ell , \end{aligned}$$

the KKT conditions are readily identified. Of particular importance is the stationarity of the Lagrangian *L* with respect to the length-orientation distribution $$f $$, i.e., the vanishing of the variation with respect to the length-orientation distribution $$f $$

2.18 $$\begin{aligned} {\varvec{0}}\,= \frac{\delta L}{\delta f}(f,{\varvec{B}},\zeta ) \equiv - \log f {(\ell ,{\varvec{p}})} + \ell \, {\varvec{B}}: {\varvec{p}}\otimes {\varvec{p}}\, + {\zeta (\ell )}, \end{aligned}$$

which leads to the following functional expression

2.19 $$\begin{aligned} f^{\texttt {MEE}}(\ell ,{\varvec{p}}) = \exp \left( \zeta (\ell ) + \ell \, {\varvec{B}}: {\varvec{p}}\otimes {\varvec{p}}\right) \end{aligned}$$

of the maximum entropy closure or the maximum entropy estimate of the length-orientation distribution. To stress that the distribution function (2.19) arises from the maximum-entropy estimate, we add a superscript $\texttt {MEE}$. The remaining two KKT conditions are given by the constraints (2.13).

2.20 $$\begin{aligned}  &   \displaystyle \int _0^\infty \!\!\int _{S^2} { \ell \, {\varvec{p}}\otimes {\varvec{p}}\, f {(\ell ,{\varvec{p}})}} \, dS \,d\ell \nonumber \\  &   \quad = \bar{\ell }\,{\varvec{A}}  \quad \text {and} \quad \displaystyle \int _{S^2} {f (\ell ,{\varvec{p}})} \, dS = \psi  (\ell ), \quad \ell >0, \end{aligned}$$

In view of the Bingham distribution (2.10), we may re-write Eq. (2.19) in the form

2.21 $$\begin{aligned} \begin{aligned} f^{\texttt {MEE}}(\ell ,{\varvec{p}})&= \exp \left( \zeta (\ell ) + \ell \, {\varvec{B}}: {\varvec{p}}\otimes {\varvec{p}}\right) \\&= \exp \left( \zeta (\ell ) + c(\ell \,{\varvec{B}}) + \ell \, {\varvec{B}}: {\varvec{p}}\otimes {\varvec{p}}- c(\ell \,{\varvec{B}})\right) \\&= \exp \left( \zeta (\ell ) + c(\ell \,{\varvec{B}})\right) \exp \left( \ell \, {\varvec{B}}: {\varvec{p}}\otimes {\varvec{p}}- c(\ell \,{\varvec{B}})\right) \\&= \exp \left( \zeta (\ell ) + c(\ell \,{\varvec{B}})\right) \varphi ^\texttt {Bingham}_{\ell \, {\varvec{B}}}({\varvec{p}}) \\ \end{aligned} \end{aligned}$$

in terms of the normalization constant $c(\ell \, {\varvec{B}})$ of the Bingham distribution (2.11). The second equation in the constraints (2.20) provides the expression

2.22 $$\begin{aligned} f^{\texttt {MEE}}(\ell ,{\varvec{p}}) = \psi  (\ell )\,\varphi ^\texttt {Bingham}_{\ell \, {\varvec{B}}}({\varvec{p}}) \end{aligned}$$

for the length-orientation distribution function. In particular, we observe that length and orientation are coupled via the parameter ${\varvec{M}}= \ell \, {\varvec{B}}$ of the Bingham distribution.

A few remarks are in order.

1. If the fiber-orientation tensor ${\varvec{A}}$ is isotropic, it follows that ${\varvec{B}}= 0$.
2. As the isotropic fiber-orientation state is described by the Bingham parameter ${\varvec{M}}= 0$, the functional relationship ${\varvec{M}}= \ell \, {\varvec{B}}$ in the Bingham parameter specifies that shorter fibers ($\ell \ll \bar{\ell }$) are *more isotropic* than longer fibers ($\ell \gg \bar{\ell }$). Generally, the latter case approaches either a uni-directional or a planar isotropic orientation state as $\ell \rightarrow \infty$. We will take a closer look at these effects in the computational-experiments Sect. 4.3.
3. If we considered the constraint
  2.23 $$\begin{aligned} \displaystyle \int _0^\infty \!\!\int _{S^2} { {\varvec{p}}\otimes {\varvec{p}}\, f } \, dS \,d\ell = {\varvec{A}} , \end{aligned}$$
  where fiber lengths are ignored, instead of the length-weighted constraint (2.13), the corresponding maximum-entropy length-orientation closure would be uncoupled (2.14). In turn, the uncoupled model (2.14) may be associated to the (implicit) assumption (2.23).
4. The outlined procedure works in the case that the fiber-orientation tensor ${\varvec{A}}$ is non-degenerate, i.e., has a non-vanishing determinant. If the degenerate case appears in practice, we perturb the orientation tensor slightly to make it non-degenerate.

## Generating fiber-filled volume elements with maximum-entropy length-orientation closure

### Algorithmic overview

**Algorithm 1:**
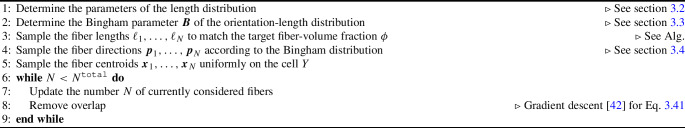
Algorithmic overview of the steps with inputs described in section 3.1

Before discussing the microstructure-generation algorithm, it is essential to define both the inputs and the output(s) of the procedure precisely. For a start, we suppose the following items to be provided:

- A fiber-length distribution $\psi$ with identified parameters, either defined explicitly identified inversely based on certain targeted statistical quantities. In case of the latter scenario and where the number-weighted mean and standard deviation are prescribed, details on the identification procedure for commonly used distribution functions are discussed in Sect. 3.2.
- A prescribed second-order fiber-orientation tensor ${\varvec{A}}$, i.e., a second order symmetric and positive semi-definite tensor with unit trace.
- The fiber diameter *D*.
- The target volume fraction $\phi \in (0,1)$.
- The (periodic) unit cell $Y = [0,Q_1] \times [0,Q_2] \times [0,Q_3]$.

The target is to generate a number ${N^{\texttt {total}}}$ of fibers with centroids ${\varvec{x}}_i \in Y$, directions ${\varvec{p}}_i \in S^2$ and lengths, such that the described cylinders are in a non-overlapping configuration, their volume fraction matches $\phi$, the length-weighted fiber-orientation tensor of fourth order

3.1 $$\begin{aligned} \mathbb {A} _N \equiv \frac{1}{\bar{\ell }_N} \sum _{i=1}^N \ell _i {\varvec{p}}_i \otimes {\varvec{p}}_i \otimes {\varvec{p}}_i \otimes {\varvec{p}}_i, \quad \bar{\ell }_N \equiv \frac{1}{N} \sum _{i=1}^N \ell _i, \end{aligned}$$

is sufficiently close to the prescribed fourth-order fiber-orientation tensor

3.2 $$\begin{aligned} \mathbb {A}  = \frac{1}{\bar{\ell }} \displaystyle \int _0^\infty \!\!\int _{S^2} { \ell \, {\varvec{p}}\otimes {\varvec{p}}\otimes {\varvec{p}}\otimes {\varvec{p}}\, f^{\texttt {MEE}}(\ell ,{\varvec{p}})} \, dS \,d\ell \end{aligned}$$

corresponding to the maximum-entropy closure estimate (2.22), together with certain statistical moments of the length distribution

3.3 $$\begin{aligned} \frac{1}{N}\sum _{i=1}^N \ell _i^m \approx \displaystyle \int _0^\infty {\ell ^m} \, d\ell , \quad m=1,2,\ldots \end{aligned}$$

It is implicitly assumed that this problem is actually solvable, i.e., that the cell *Y* is sufficiently large and that the volume fraction $\phi$ is small enough. Of course, whether the problem is solvable or not, and whether *the presented algorithm* is able to produce such a sought solution is a non-trivial matter, and we will confine ourselves to empirical checks on the convergence of the algorithm.

An overview of the actual workflow from the given quantities to the desired realization of the fiber-filled microstructure is shown in Algorithm 1. There is a number of preprocessing steps which are required to produce both the initial configuration required for the modified SAM algorithm, see Sect. 3.5, and the targeted fourth-order orientation tensor (3.2), resulting from the maximum-entropy length-orientation closure $f^{\texttt {MEE}}$, see Eq. (2.22). We will discuss the procedure to obtain the parameters of a selected fiber-length distribution, such that the number-weighted or volume-weighted mean and standard deviation are matched, first, see Sect. 3.2. Once the fiber-length distribution is known, the next step involves computing the Bingham parameter ${\varvec{B}}\in \text {Sym}_0(3)$ solving (right side of) the equation (2.20), see Sect. 3.3 for details. Sampling the initial configuration is discussed in Sect. 3.4, and an outline of the SAM algorithm comprises Sect. 3.5.

### Identifying the length distribution

We suppose that a fixed fiber-length distribution $\psi$ is given which depends on *K* parameters. However, the values of the *K* parameters are not known. Instead, only the first (nontrivial) *K* moments of the distribution are known. Then, it is often possible to identify the parameters inversely by matching the prescribed moments.

For the manuscript at hand, we consider only the case with $K=2$ parameters and where the prescribed moments are *length-weighted*. In fact, for fiber composites with uniform diameter, statistics are typically obtained based on *volume averages*, leading to length-weighted moments in a natural way [45, §2.2].

To reduce the notational burden, we denote the expectation of a random variable *Z* with respect to the fiber-length distribution $\psi$ by angular brackets

3.4 $$\begin{aligned} \left\langle {Z} \right\rangle \equiv \displaystyle \int _0^\infty {Z(\ell ) \, \psi  (\ell )} \, d\ell . \end{aligned}$$

We suppose that both a volume-weighted mean $\mu _{\ell }$ and a volume-weighted standard deviation $\sigma _{\ell }$ are given. Then, we would like to identify the parameters of the fiber-length distribution, such that the volume-weighted mean $\mu _{\ell }$ and the volume-weighted standard deviation $\sigma _{\ell }$ *or* the number-weighted mean $\mu _{\#}$ and the number weighted standard deviation $\sigma _{\#}$ are matched *exactly*, i.e., the equations

3.5 $$\begin{aligned} \mu _{\ell } {\mathop {=}\limits ^{!}} \frac{\left\langle {\ell ^2} \right\rangle }{\left\langle {\ell } \right\rangle } \quad \text {and} \quad \sigma _{\ell }^{2} {\mathop {=}\limits ^{!}} \frac{\left\langle {(\ell - m)^2 \ell } \right\rangle }{\left\langle {\ell } \right\rangle } \end{aligned}$$

or in terms of number weighted means and standard deviations,

3.6 $$\begin{aligned} \mu _{\#} {\mathop {=}\limits ^{!}} \left\langle {\ell } \right\rangle \quad \text {and} \quad \sigma _{\#}^{2} {\mathop {=}\limits ^{!}} \left\langle {\ell ^2} \right\rangle -\left\langle {\ell } \right\rangle ^2 \end{aligned}$$

should be satisfied. Straightforward algebraic manipulations [45, Apx. A] lead to the equivalent conditions

3.7 $$\begin{aligned} \mu _{\ell } {\mathop {=}\limits ^{!}} \frac{\left\langle {\ell ^2} \right\rangle }{\left\langle {\ell } \right\rangle } \quad \text {and} \quad \sigma _{\ell }^{2} {\mathop {=}\limits ^{!}} \frac{\left\langle {\ell ^3} \right\rangle }{\left\langle {\ell } \right\rangle } - \mu _{\ell }^{2}. \end{aligned}$$

More often than not, this system of equations may be solved for the two unknown parameters of the fiber-length distribution.

To illustrate the idea let us consider the lognormal distribution [66]

3.8 $$\begin{aligned} \psi ^{\texttt {log}}(\ell ) = \frac{1}{\ell \, \sigma _{\text {ln}}\, \sqrt{2\pi }} \, \exp \left( - \frac{(\ln \ell /\ell _0)^2}{2\sigma _{\text {ln}}^2} \right) , \quad \ell > 0, \end{aligned}$$

which involves two parameters, $\ell _0$ with dimension of lengths and the non-dimensional standard deviation $\sigma _{\text {ln}}$. Notice that the probability distribution (3.8) has the correct dimension one over length, such that its mean with respect to $d\ell$ equals (the dimension-less) unity. To proceed, we take a look at explicit expressions for the moments [66, §2.3]

3.9 $$\begin{aligned} \left\langle {\ell ^\alpha } \right\rangle = \ell _0^\alpha \, e^{\alpha ^2 \sigma _{\text {ln}}^2 /2} \quad \text {for } \quad \alpha =0,1,2,\ldots \end{aligned}$$

Inserting these into the Eq. (3.7) yields the expressions

3.10 $$\begin{aligned} \ell _0 \, e^{3 \sigma _{\text {ln}}^2 /2} = \mu _{\ell } \quad \text {and} \quad \ell _0^2 \, e^{4 \sigma _{\text {ln}}^2} - \mu _{\ell }^{2} = \sigma _{\ell }^{2}. \end{aligned}$$

and similarly from equation (3.6) it follows,

3.11 $$\begin{aligned} \ell _0 \, e^{\sigma _{\text {ln}}^2 /2} = \mu _{\#} \quad \text {and} \quad \ell _0^2 \, e^{2 \sigma _{\text {ln}}^2} - \mu _{\#}^{2} = \sigma _{\#}^{2}. \end{aligned}$$

Thus, Eqs. (3.10) and (3.11) yields

3.12 $$\begin{aligned} \ell _0 = \mu _{\ell } \, e^{-3 \sigma _{\text {ln}}^2 /2} = \mu _{\#} \, e^{- \sigma _{\text {ln}}^2 /2}, \end{aligned}$$

which might be inserted into the second equation to provide the relation

3.13 $$\begin{aligned}  &   e^{ \sigma _{\text {ln}}^2} - 1 = \frac{\sigma _{\ell }^{2}}{\mu _{\ell }^{2}} = \frac{\sigma _{\ell }^{2}}{\mu _{\#}^{2}}, \quad \text {i.e.,} \quad \sigma _{\text {ln}}\nonumber \\  &   \quad = \sqrt{\log ( 1 + \sigma _{\ell }^{2} / \mu _{\ell }^{2})} = \sqrt{\log ( 1 + \sigma _{\#}^{2} / \mu _{\#}^{2})}. \end{aligned}$$

In turn, the reference length $\ell _0$ computes as

3.14 $$\begin{aligned} \ell _0 = \mu _{\ell } \, \left( 1 + \frac{\sigma _{\ell }^{2}}{\mu _{\ell }^{2}}\right) ^{-3/2} = \mu _{\#} \, \left( 1 + \frac{\sigma _{\#}^{2}}{\mu _{\#}^{2}}\right) ^{-1/2}, \end{aligned}$$

Other length distributions, e.g., the $\Gamma$-distribution [67], admit similar explicit expressions. However, there are distributions where iterative methods appear to be imperative when determining the necessary parameters like the Weibull distribution [45, §2.2].

### Determining the Bingham parameter

In Sect. 2.2 we derived an expression for the orientation-length distribution function (2.22)

3.15 $$\begin{aligned} f^{\texttt {MEE}}(\ell ,{\varvec{p}}) = \psi  (\ell )\,\varphi ^\texttt {Bingham}_{\ell \, {\varvec{B}}}({\varvec{p}}) \end{aligned}$$

predicted by the maximum-entropy estimate (2.22), where ${\varvec{B}}\in \text {Sym}_0(3)$ denotes a traceless and symmetric $3 \times 3$-matrix which is determined by the first constraint in equation (2.20)

3.16 $$\begin{aligned} \displaystyle \int _0^\infty \!\!\int _{S^2} { \ell \, {\varvec{p}}\otimes {\varvec{p}}\, f ^{\texttt {MEE}}{(\ell ,p)}} \, dS \,d\ell = \bar{\ell }\,{\varvec{A}} . \end{aligned}$$

As the fiber-length distribution is fixed and known, see Sect. 3.2, the Bingham parameter ${\varvec{B}}\in \text {Sym}_0(3)$ must be determined in the next step.

For a start, we notice the identity

3.17 $$\begin{aligned}  &   \frac{\partial c}{\partial {\varvec{B}}} ({\varvec{B}}) = \displaystyle \int _{S^2} {{\varvec{p}}\otimes {\varvec{p}}\, \varphi ^\texttt {Bingham}_{{\varvec{B}}}({\varvec{p}})} \, dS, \nonumber \\  &   \quad \text {valid for any} \quad {\varvec{B}}\in \text {Sym}_0(3), \end{aligned}$$

which follows from the definition (2.11) of the normalizing constant. For later use, we also record the identity

3.18 $$\begin{aligned}  &   \frac{\partial ^2 c}{\partial {\varvec{B}}^2} ({\varvec{B}}) = \displaystyle \int _{S^2} {{\varvec{p}}\otimes {\varvec{p}}\otimes {\varvec{p}}\otimes {\varvec{p}}\, \varphi ^\texttt {Bingham}_{{\varvec{B}}}({\varvec{p}})} \, dS, \nonumber \\  &   \quad \text {valid for any} \quad {\varvec{B}}\in \text {Sym}_0(3). \end{aligned}$$

Using the identity (3.17), we may rewrite condition (3.16) in the form

3.19 $$\begin{aligned} \bar{\ell }\,{\varvec{A}}  = \displaystyle \int _0^\infty { \ell \, \displaystyle \int _{S^2} {{\varvec{p}}\otimes {\varvec{p}}\, f ^{\texttt {MEE}}{(\ell ,p)}} \, dS} \, d\ell . \end{aligned}$$

Thus, the Bingham parameter ${\varvec{B}}\in \text {Sym}_0(3)$ is determined from the condition

3.20 $$\begin{aligned} \frac{1}{\bar{\ell }}\, \displaystyle \int _0^\infty { \frac{\partial c}{\partial {\varvec{B}}} (\ell \, {\varvec{B}}) \, \ell \,\psi  (\ell )} \, d\ell = {\varvec{A}}  \end{aligned}$$

for prescribed second-order fiber-orientation tensor ${\varvec{A}}$.

To solve the condition (3.20) on a computer, it appears necessary to approximately evaluate the integral in question. In fact, for a given fiber-length distribution $\psi$, we suppose an approximation by quadrature

3.21 $$\begin{aligned} \psi  (\ell ) \, d\ell \approx \sum _{q=1}^Q w_q \, \delta (\ell - \ell _q) \end{aligned}$$

with *Q* quadrature lengths $\ell _q$, suitable non-negative weights $w_q$ and the Dirac distribution $\delta$ at zero, is given. Details on designing an appropriate quadrature rule are given in Sect. 3.4.

In the approximation by quadrature (3.21), the condition (3.20) becomes

3.22 $$\begin{aligned} \sum _{i=q}^Q \frac{w_q \, \ell _q}{\bar{\ell }} \, \frac{\partial c}{\partial {\varvec{B}}} (\ell _q \, {\varvec{B}}) = {\varvec{A}} . \end{aligned}$$

This equation may be solved by Newton’s method [68, §9.5],

3.23 $$\begin{aligned} {\varvec{B}}\leftarrow {\varvec{B}}+ s\, \triangle {\varvec{B}}, \end{aligned}$$

where the increment $\triangle {\varvec{B}}$ is determined by the linear equation

3.24 $$\begin{aligned} \sum _{q=1}^Q \frac{w_q \, \ell _q^2}{\bar{\ell }} \, \frac{\partial ^2 c}{\partial {\varvec{B}}^2} (\ell _q \, {\varvec{B}}):\triangle {\varvec{B}}= {\varvec{A}}  - \sum _{q=1}^Q \frac{w_q \, \ell _q}{\bar{\ell }} \, \frac{\partial c}{\partial {\varvec{B}}} (\ell _q \, {\varvec{B}}) \end{aligned}$$

and $s \in (0,1]$ is a backtracking factor.

In practice, it is convenient to diagonalize the tensors ${\varvec{A}}$ and ${\varvec{B}}$ jointly, to reorder the eigenvalues $b_i$ of ${\varvec{B}}$ to $b_1 \le b_2 \le b_3$ and to eliminate one of the eigenvalues via the constraint $0=b_1 + b_2 + b_3$. Then, only a $2 \times 2$ linear system needs to be solved in every Newton step.

Moreover, due to the the identities (3.17) and (3.18), the quantities $\partial c / \partial {\varvec{B}}$ and $\partial ^2 c / \partial {\varvec{B}}^2$ required in Newton’s method (3.24) correspond to second and fourth order moments of the Bingham distribution. Efficient and accurate implementations are available for computing these moments. We rely upon the implementation provided alongside the article by Luo et al. [69].

Notice that the strict convexity of the entropy functional (2.15), the minimizing distribution $$f $$ is unique. Due to the specific form (2.22) of the minimizer, the Lagrange multiplier $${\varvec{B}}$$ is unique up to the addition of a multiple of the identity. By fixing the trace of the tensor $${\varvec{B}}$$ to zero, solutions $${\varvec{B}}\in \text {Sym}_0(3)$$, i.e., with trace zero, to the Eq. (3.16) are unique, as well.

### Length and orientation sampling

**Algorithm 2:**
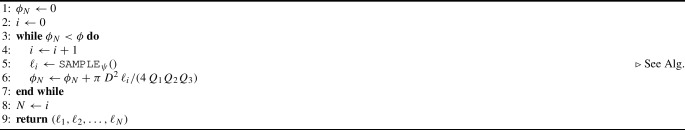
Sampling the length ($\phi$,*D*,*Y*,$\psi$)

**Algorithm 3:**
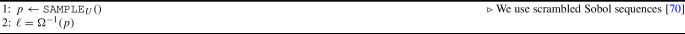
PPF-based length sampler $\texttt {SAMPLE}_{\psi  }$ using a sampler $\texttt {SAMPLE}_{{U}}$ for $\mathcal {U}([0,1])$

Once the fiber-length distribution $$\psi  $$ is identified, together with the Bingham parameter $${\varvec{B}}\in \text {Sym}_0(3)$$ entering the maximum-entropy expression (2.22) for the length-orientation distribution function $$f $$, the next task consists of drawing fibers with centroids $${\varvec{x}}_i \in Y$$, orientations $${\varvec{p}}_i \in S^2$$ and lengths $$\ell _i > 0$$.

There are different ways to determine the number ${N^{\texttt {total}}}$ of fibers to be drawn. Suppose that we draw ${N^{\texttt {total}}}$ fibers with lengths $\ell _1,\ldots ,\ell _N$. In a non-overlapping configuration, the resulting fiber-volume fraction $\phi _N$ computes as

3.25 $$\begin{aligned} \phi _N = \left. \frac{\pi D^2}{{4}} \sum _{i=1}^N \ell _i \Bigg {/} \text {vol}(Y) \right. , \end{aligned}$$

where *D* refers to the fiber diameter and $\text {vol}(Y) = Q_1 Q_2 Q_3$ stands for the volume of the unit cell $Y=[0,Q_1] \times [0,Q_2] \times [0,Q_3]$. A strategy would be to replace the total length by $N \bar{\ell }$, leading to the estimate

3.26 $$\begin{aligned} N^{\texttt {total}} \approx \frac{4}{\pi } \, \frac{\text {vol}(Y)}{\bar{\ell } D^2 } \, \phi \end{aligned}$$

for the total number of fibers to be drawn. However, this estimate suffers from the error in the sampled mean length compared to the ensemble mean length $\bar{\ell }$, which may be rather large for small samples. Thus, we opt for an adaptive strategy where fiber lengths are drawn successively until the targeted volume fraction is exceeded. All in all, we are led to the Algorithm 2, where we assume that there is some sampling procedure $\texttt {SAMPLE}_{\psi  }()$ for the distribution $\psi$ available.

In addition to matching the volume fraction, we would like the drawn fiber lengths $\ell _1,\ldots ,\ell _N$ to resemble the continuous distribution $\psi$ as close as possible. For instance, the empirical moments and the continuous moments should be close

3.27 $$\begin{aligned} \frac{1}{N} \sum _{i=1}^N \ell _i^\alpha \approx \displaystyle \int _0^\infty {\ell ^\alpha } \, d\ell \end{aligned}$$

for relevant exponents $\alpha \in \mathbb {N}$. To understand such a moment-matching problem better, it is convenient to regard the problem (3.27) as a *quadrature rule* with uniform weights $w_i = 1/N$. Then, one observes that a random sampling leads to an error for the approximation (3.27) that scales as $N^{-1/2}$. Put differently, to reduce the quadrature error by one order of magnitude, 100 times as many samples need to be drawn. As a particular consequence, random sampling does not lead to high accuracy.

To mitigate the problem, quasi-random sampling strategies were introduced [71, 72], which are based on a deterministic sequence $x_1,x_2,\ldots$ of points in the unit cube, such that the integration error decreases as $N^{-1}$ for sufficiently smooth integrands, up to a logarithmic factor that depends on the spatial dimension. With such a favorable scaling, only ten times as many samples as required to reduce the error by an order of magnitude, on average. Moreover, in case the integrand is sufficiently smooth, an additional randomization strategy, called scrambling [70], reduces the error decay to $N^{-3/2}$.

These quasirandom strategies are typically designed for the uniform distribution on the unit cube, i.e., on the unit interval for one-dimensional problems. To apply such a strategy to problems of the form (3.27), it is convenient to use a suitable transformation of coordinates. More precisely, suppose we wish to approximate the integral

3.28 $$\begin{aligned} I(Z) = \displaystyle \int _0^\infty {Z(\ell )\psi (\ell )} \, d\ell \end{aligned}$$

for a random variable $Z:(0,\infty ) \rightarrow \mathbb {R}$ by an empirical quadrature

3.29 $$\begin{aligned} I_N(Z) = \frac{1}{N} \sum _{i=1}^N Z(\ell _i). \end{aligned}$$

We introduce the cumulative distribution function

3.30 $$\begin{aligned} \Omega  : (0,\infty ) \rightarrow (0,1), \quad \ell \mapsto \int _0^\ell \psi (\tilde{\ell }) \, d\tilde{\ell }. \end{aligned}$$

In case the length-distribution function $\psi$ is continuous and positive, the cumulate length-distribution function $\Omega$ is strictly monotone and continuously differentiable with derivative $\Omega  ' = \psi$. Due to the strict monotonicity, the cumulative distribution function $\Omega$ is actually a bijection, i.e., admits an inverse $\Omega  ^{-1}:(0,1) \rightarrow (0,\infty )$. With these notions at hand, we may use the mapping $q = \Omega (\tilde{\ell })$ to transform the integral (3.28)

3.31 $$\begin{aligned} I(Z) \equiv \displaystyle \int _0^\infty {Z(\ell )\psi (\ell )} \, d\ell = \int _0^1 Z(\Omega  ^{-1}(q)) \, dq, \end{aligned}$$

where we used $dq = \Omega ' \, d\tilde{\ell } \equiv \psi (\tilde{\ell }) \, d\tilde{\ell }$. For a given quadrature rule with points $q_i \in (0,1)$ and weigths $w_i$ on the unit interval, we may thus construct a quadrature rule for the integral (3.28) via

3.32 $$\begin{aligned} I(Z) \approx \sum _{i=1}^N w_i \, Z(\Omega  ^{-1}(q_i)). \end{aligned}$$

Thus, in case both the random variable *Z* and the inverse of the cumulative distribution function $\Omega$ are sufficiently regular, we may use quasirandom numbers $q_i$ to obtain length samples $\ell _i = \Omega  ^{-1}(q_i)$ such that the empirical approximations (3.29) approximate the integrals (3.28) with a rate 1/*N*. If scrambled quasi-random numbers are used, the rate improves to $N^{-3/2}$. The procedure is summarized in Algorithm 3.

**Fig. 3:**
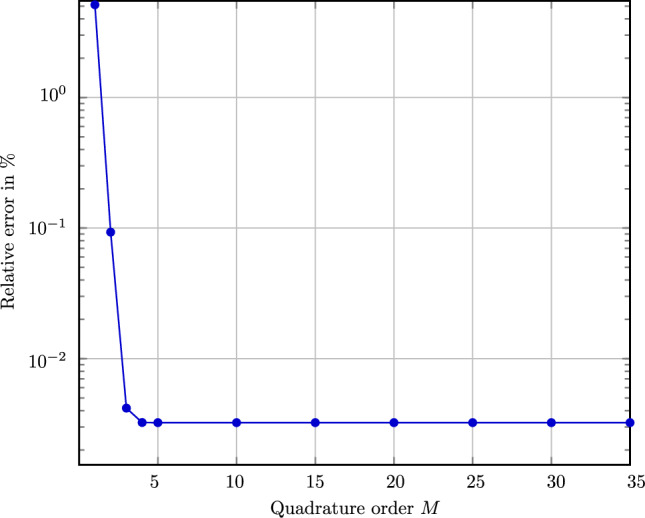
Relative error in empirical mean fiber length from Gauss-Hermite quadrature rule for $\mu _{\#} = 300\upmu$m and $\sigma _{\#} = 100\upmu$m, depending on the number of quadrature points *M*

There is a second use of the transformation rule (3.32), namely to provide a highly accurate quadrature rule (3.21) for use in the Eq. (3.22) determining the Bingham parameter ${\varvec{B}}\in \text {Sym}_0(3)$. In this case, the number of quadrature points is actually independent of the fiber count, and we will use the letter *M* to designate this quadrature-point count. In our implementation, we use the Gauss- Hermite quadrature [73, Eq. (25.4.46)] of degree 35, which is available in scipy [74]. Actually, using degree 35 is more than sufficient for the applications at hand. To illustrate this statement, consider drawing samples from a lognormal distribution (3.8) with parameters $\mu _{\#} = 300~\upmu$m and $\sigma _{\#} = 100~\upmu$m, which we determine from (3.11). In particular, we may use the prescribed parameters to assess the accuracy of the sampling. More precisely, we study the empirical mean (3.27) of the considered length distribution function $\psi  ^{\texttt {log}}$ and monitor the relative deviation with respect to the prescribed mean $\mu _{\#}$, see Fig. 3. We observe a rapid decrease of the relative error up to about $M=5$ quadrature points, reaching a relative error less than $0.01\%$. Higher quadrature order does not improve this level of accuracy. However, the realized order of magnitude in error appears sufficient for engineering purposes. In any case, using $M=35$ quadrature points permits us to use the Gauss-Hermite quadrature with confidence. Moreover, implementations of the inverse cumulative length distribution $\Omega  ^{-1}$ are widely available, e.g. under the name “percent point function” in scipy.stats.

Once the fiber lengths $\ell _1,\ell _2,\ldots , \ell _N$ are identified, we sample the fiber directions ${\varvec{p}}_i$. For each index $i = 1,\ldots ,N$, the direction ${\varvec{p}}_i$ follows the Bingham distribution (2.10) with parameter ${\varvec{M}}_i = \ell _i {\varvec{B}}$. Thus, a fast and robust sampling procedure for essentially arbitrary Bingham distributions is imperative. For the work at hand, we use a rejection sampling based on the ACG distribution (2.9), as introduced by Kent et al. [75], which requires solving a single nonlinear equation.

Once both the lengths and directions are sampled, it remains to sample the centroids ${\varvec{x}}_i$ uniformly within the unit cell $Y=[0,Q_1] \times [0,Q_2] \times [0,Q_3]$. An intuitive idea would be to sample three numbers $\xi _1$, $\xi _2$ and $\xi _3$, each following the uniform distribution $\mathcal {U}([0,1])$ on the unit interval and then to use $Q_a\xi _a$ $(a=1,2,3)$ for the coordinates of the centroid. However, this naive procedure may not be optimal in case of strong differences between the edge lengths $Q_1$, $Q_2$ and $Q_3$. Indeed, the Euclidean distances between the centroids will not be uniform, but appear artificially distorted due to the rescaling by the factors $Q_a$.

This downside is readily mitigated by using a rejection sampling strategy on the cube $\left[ 0,\max _{a=1}^3 Q_a\right] ^3$, i.e., one draws ${\varvec{x}}_i$ following $\mathcal {U}\left( \left[ 0,\max _{a=1}^3 Q_a\right] ^3 \right)$ and accepts the sample provided it is admissible, i.e., the condition ${\varvec{x}}_i \in Y$ holds.

To conclude this section, let us emphasize that we use classical random sampling for both the directions ${\varvec{p}}_i$ and the centroids ${\varvec{x}}_i$. Indeed, the SAM algorithm will reduce the mismatch of the fiber-orientation state anyway, so that there is not much of a problem. For the drawn fiber lengths, significantly higher effort was required, as the number of sampled fibers does not change after this preprocessing state, excluding any possibility to correct any introduced errors later on.

We refer to Mehta and Schneider [45] for more background and details on the Weibull distribution.

### Integration into the SAM framework

The sequential addition and migration (SAM) algorithm is based on an extension of the overlap-removal technique introduced by Williams and Philipse [41] in the mechanical-contraction method (MCM). More precisely, the SAM algorithm alternates two steps. In a first step, fibers are added to the volume in a (possibly) non-overlapping configuration. In the second step, the induced overlap is removed, taking into account the desired fiber-orientation state. The SAM procedure differs from the MCM strategy in two key aspects. For MCM, all objects are introduced from the very beginning, but into a possibly very large cell. Then, the MCM algorithm alternates between shrinking the cell, increasing the volume-fraction in the process, and removing overlap. This cell-shrinking procedure was introduced to generate rather dense packings with an essentially isotropic orientation pattern. As the original overlap removal strategy does not account for the orientation at all, care had to be taken when shrinking the cell in order not to perturb the isotropy.

The SAM algorithm was designed with more general, possibly anisotropic orientation states in mind. Therefore, the fiber-orientation state, encoded by the fourth-order fiber-orientation tensor (2.5) is accounted for during the overlap removal. On the downside, the orientation state is only enforced in terms of the used orientation measure. The advantages are manifold. For a start, a much more aggressive contraction strategy is feasible than for the original MCM method [41], leading to much higher achievable volume fractions [42]. Secondly, it is not necessary to account for all fibers from the very beginning, as done for the MCM method, and to work with a large cell. Rather, the target cell can be used from the very beginning and the fibers are added in portions. The latter strategy leads to a much better performance, as the number of processed potential fiber collisions is typically much small.

The first step is implemented in a simple way. Actually, all ${N^{\texttt {total}}}$ fibers are placed in the cell *Y*, as described in Sect. 3. However, only a fraction of the fibers is actually processed, i.e., the first *N* fibers are accounted for in the overlap check with ${N} \le {N^{\texttt {total}}}$. Here *N* represents current number of fibers. Once the overlap is removed, the integer *N* is increased.

The heart of the SAM algorithm is the orientation-aware overlap-removal strategy. It is convenient to consider fibers as spherocylinders, i.e., as cylinders with spherical caps attached, for the overlap removal. The *i*-th fiber with centroid ${\varvec{x}}_i$, direction ${\varvec{p}}_i$ and length $\ell _i$, and the increased diameter $\tilde{D}>D$ is thus described by the set

3.33 $$\begin{aligned} S_i  &   = \left\{ {\varvec{x}}\in Y \, |\, \text {dist}_Y\left( {\varvec{x}}, {\varvec{x}}_i + \frac{s}{2} \, \ell _i \, {\varvec{p}}_i\right) \right. \nonumber \\  &   \left. < \frac{{\tilde{D}}}{2} \quad \text {for some} \quad s \in [-1,1]\right\} , \end{aligned}$$

where $\text {dist}_Y$ denotes the periodic distance on the rectangular cell *Y*, i.e., all points which are sufficiently close to the central line segment. Spherocylinders are used as they permit a particularly simple overlap check. Indeed, two spherocylinders $S_i$ and $S_j$ overlap precisely if the distance

3.34 $$\begin{aligned} d_{ij}  &   = \min \left\{ \text {dist}_Y\left( {\varvec{x}}_i + \frac{s}{2} \, \ell _i \, {\varvec{p}}_i, {\varvec{x}}_j + \frac{s}{2} \, \ell _j \, {\varvec{p}}_j\right) \right. \nonumber \\  &   \quad \left. | \, s_i \in [0,1], \quad s_j \in [0,1] \right\} \end{aligned}$$

between the two central line segments is smaller than the diameter ${\tilde{D}}$. Put differently, the two spherocylinders $S_i$ and $S_j$ are in a non-overlapping configuration provided the inequality

3.35 $$\begin{aligned} d_{ij} \ge {\tilde{D}} \end{aligned}$$

holds. Algebraic manipulations show that the condition (3.35) is equivalent to the equation

3.36 $$\begin{aligned} \delta _{ij} {\mathop {=}\limits ^{!}}0 \end{aligned}$$

with the constraint qualifier

3.37 $$\begin{aligned} \delta _{ij} = \max \left( 0, {\tilde{D}} - \delta _{ij}\right) . \end{aligned}$$

As the quantity $\delta _{ij}$ is non-negative, all fibers are in a non-negative configuration provided the condition

3.38 $$\begin{aligned} \sum _{i<j} \delta _{ij}^2 {\mathop {=}\limits ^{!}} 0 \end{aligned}$$

holds. Moreover, we enforce the condition

3.39 $$\begin{aligned} \mathbb {A} _N {\mathop {=}\limits ^{!}} \mathbb {A} , \end{aligned}$$

i.e., the empirical fiber-orientation tensor

3.40 $$\begin{aligned} \mathbb {A} _N = \frac{1}{N \, \bar{\ell }_N} \sum _{i=1}^{N} \ell _i {\varvec{p}}_i^{\otimes 4} \quad \text {with} \quad \bar{\ell }_N = \sum _{i=1}^{N} \ell _i \end{aligned}$$

should match the prescribed fiber-orientation tensor (2.5), which arises from the length-orientation closure (2.22). We introduce the objective function

3.41 $$\begin{aligned} R({\varvec{x}}_1,\ldots ,{\varvec{x}}_N,{\varvec{p}}_1,\ldots ,{\varvec{p}}_N) = \frac{1}{2} \sum _{i<j} \delta _{ij}^2 + \frac{\bar{\lambda }}{8} \Vert \mathbb {A}  - \mathbb {A} _N\Vert ^2 \end{aligned}$$

encoding the overlap removal with the prefactor

3.42 $$\begin{aligned} \bar{\lambda } = \frac{{\tilde{D}}^2}{ {2}/{3} + \bar{r}_a} \left[ \frac{\bar{r}_a^{\,3}}{12} + \frac{\bar{r}_a^{\,2}}{6} + \frac{3 \, \bar{r}_a}{16} + \frac{1}{15} \right] , \end{aligned}$$

involving the mean aspect ratio $\bar{r}_a = \bar{\ell }/{\tilde{D}}$.

The function *R* in Eq. (3.41) is zero precisely if the non-overlapping configuration condition (3.35) and the fiber-orientation constraint (3.39) are satisfied. Moreover, the function (3.41) is continuously differentiable. The SAM algorithm uses the associated gradient-descent method with a suitable step size to find a configuration with sufficiently small value of the function *R*, see Schneider [42] for details.

Notice that a minimum inter-fiber distance is easily accounted for in the algorithm by increasing the fiber diameter ${\tilde{D}}$ artificially for the overlap checks. Details on the efficient implementation can be found in Schneider [42], and we refer to Mehta and Schneider [45] for special considerations required for handling long fibers.

Once the final fiber microstructure is obtained, the fiber caps are removed, and only the cylinders are voxelated.

## Computational investigations

### Setup

We implemented the maximum-entropy closure approximation, see Eq. (2.13), into an existing *serial* fiber generator [42, 45] implemented in Python with Cython extension. We made use of the Algorithm [69] for computing the moments of the Bingham distribution (2.9), more precisely, we used the implementation provided with the article which was written in the $\texttt {C}$ programming language.

**Table 1:** Isotropic elastic moduli of matrix as well as fibers (left) and typical properties of the unit cells for the glass-fiber reinforced PBT [3]

Material	*E* in GPa	$\nu$	Fiber volume fraction	$13.0\%$
E-glass fibers	73.0	0.22	Mean fiber length $\mu _{\#}$	$235.47\,\upmu$m
PBT matrix	1.70	0.35	Fiber diameter	$12.70\,\upmu$m
			Length distribution	Lognormal

**Table 2:** Isotropic elastic moduli of matrix as well as fibers (left) and typical properties of the unit cells for the glass-fiber reinforced polyamide [76]

Material	*E* in GPa	$\nu$	Fiber volume fraction	$19.3\%$
E-glass fibers	72.0	0.22	Mean fiber length $\mu _{\#}$	$275.70\,\upmu$m
PA6.6 matrix	3.0	0.4	Fiber diameter	$10\,\upmu$m
			Length distribution	Weibull

For a fixed unit cell $Y=[0,Q_1] \times [0,Q_2] \times [0,Q_3]$ with given stiffness distribution $\mathbb {C}$ on *Y*, the apparent elastic stiffness $\mathbb {C}^{\texttt {app}}$ arises as follows. For prescribed macroscopic strain $\overline{\varvec{\varepsilon }}$, we seek the periodic displacement fluctuation field ${\varvec{u}}_{\overline{\varvec{\varepsilon }}}:Y \rightarrow \mathbb {R}^3$ which solves the balance equation

4.1 $$\begin{aligned} \text {div }\mathbb {C}:(\overline{\varvec{\varepsilon }} + \nabla ^s {\varvec{u}}_{\overline{\varvec{\varepsilon }}}) = 0 \end{aligned}$$

on the unit cell *Y*. The corresponding apparent stress is defined by

4.2 $$\begin{aligned} \varvec{\sigma }_{\overline{\varvec{\varepsilon }}} = \frac{1}{Q_1 Q_2 Q_3} \int _Y \mathbb {C}:(\overline{\varvec{\varepsilon }} + \nabla ^s {\varvec{u}}_{\overline{\varvec{\varepsilon }}}) \, d{\varvec{x}}. \end{aligned}$$

Subsequently, the apparent stiffness $\mathbb {C}^{\texttt {app}}$ is determined from the equation

4.3 $$\begin{aligned} \mathbb {C}^{\texttt {app}} : \overline{\varvec{\varepsilon }} = \varvec{\sigma }_{\overline{\varvec{\varepsilon }}} \end{aligned}$$

for six linearly indendent strain-load cases, typically chosen as uniaxial strain loading in the three coordinate directions and three shear tests.

To discretize and solve Eq. (4.1) on a regular grid, we employ FFT-based computational micromechanics solvers [77, 78], relying upon an in-house code written in Python with Cython extensions [79]. We used the discretization on a staggered grid [80], relied upon the conjugate gradient method [81–83] for resolving the linear systems, and terminated the iterations in case the convergence criterion [84, §3.6] was lower than the prescribed tolerance $\texttt {tol} = 10^{-5}$. For the convenience of the reader, a short description of FFT-based solvers was added to Appendix 1.

The timings both for the microstructure generation and the subsequent computation of the apparent properties were recorded on a PC with a six-core Intel i7 CPU with 32GB RAM.

We will work with two material systems. For a start, we consider a commercially available polybutylene terephthalate (PBT), reinforced with glass fibers, following Müller [2, 3]. Table 1 contains the corresponding elastic moduli and the identified microstructure characteristics.

Moreover, we will investigate a glass-fiber reinforced polyamide 6.6, where experimental data both for the mechanical properties and for the length-orientation distribution is available [76], see Table 2.

To ensure confidence in the computational results, a resolution study and an RVE study, where RVE stands for representative volume element [30, 85, 86], are required. These studies are necessary to ensure both a sufficiently fine mesh and a sufficiently large considered cell [87, 88]. We refer to Müller et al. [2] for these studies corresponding to the PBT material system and to Mehta and Schneider [45] for the PA6.6 system. The studies revealed that a voxel resolution of two micron yields sufficiently accurate result, whereas the standard deviations of the apparent properties are rather small, even for comparatively small volume elements, and the systematic error is even smaller.

### The necessary size of a representative volume element

For materials with a random microstructure, the effective mechanical properties emerge only on representative volume elements, i.e., unit cells which are so large that they are typical for the statistics of the entire material and for which the applied boundary conditions do not have an impact on the results [85, 86]. In numerical practice, however, the considered units cells are necessarily finite, and the computed—so-called *apparent* properties are still to some degree random.

**Table 3:** Orthotropic Young’s moduli (mean ± standard deviation for ten runs) for RVE study with (volume–weighted) mean fiber length $\mu _{\#} = 250 \upmu$m and standard deviation $\sigma _{\#} = 100\upmu$m

Orientation	$Q$ in $\upmu$m	$E_1$ in GPa	$E_2$ in GPa	$E_3$ in GPa
iso	300	$5.797 \pm 0.014$	$5.803 \pm 0.019$	$5.791 \pm 0.012$
	600	$5.795 \pm 0.013$	$5.799 \pm 0.005$	$5.802 \pm 0.009$
	900	$5.797 \pm 0.004$	$5.799 \pm 0.003$	$5.797 \pm 0.004$
piso	300	$6.826 \pm 0.025$	$6.833 \pm 0.036$	$5.150 \pm 0.007$
	600	$6.830 \pm 0.014$	$6.840 \pm 0.008$	$5.141 \pm 0.002$
	900	$6.829 \pm 0.005$	$6.832 \pm 0.006$	$5.139 \pm 0.001$
ud	300	$13.639 \pm 0.035$	$4.813 \pm 0.017$	$4.811 \pm 0.017$
	600	$13.425 \pm 0.019$	$4.811 \pm 0.004$	$4.812 \pm 0.005$
	900	$13.412 \pm 0.010$	$4.810 \pm 0.002$	$4.810 \pm 0.003$

To assess the degree to which the considered cell is representative, we follow a statistical approach pioneered by Kanit et al. [30] and later on put on a mathematically firm ground by Gloria and Otto [89]. In fact, there are two quantities to consider. The dispersion (or random error) measures the standard deviation of the computed apparent properties on unit cells of fixed size. The bias (or systematic error) quantifies the deviation between the mean apparent properties and the effective properties. To assess the representativity, we thus monitor both the empirical variance of the computed apparent properties and the change of the mean apparent properties for increasing unit-cell size.

Previous studies [32, 42, 45, 90] have shown that both the random and the systematic error are minimal if periodized volume elements [31] are used together with periodic boundary conditions for the displacement field, see also the recent works  [91–93]. We wish to confirm these findings for the case at hand.

**Fig. 4:**
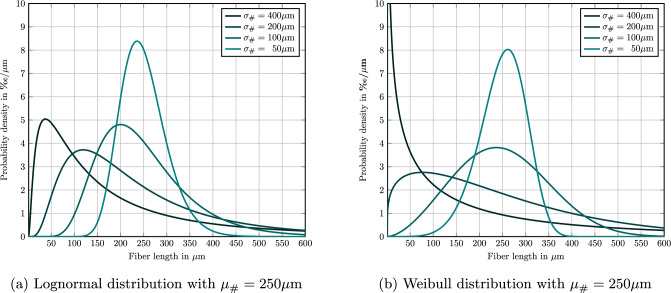
Effect of changing the standard deviation $\sigma _{\#}$ at fixed mean $\mu _{\#}$, both number weighted

We consider the Weibull fiber-length distribution [45, §2.2] with mean fiber fiber length $\mu _{\#} =250\,\upmu$m and a standard deviation $\sigma _{\#} = 100\,\upmu$m, see Fig. 4b. For the extreme second-order fiber-orientation tensors

4.4 $$\begin{aligned} {\varvec{A}}^{\texttt {iso}} =  &   \text {diag}(1/3,1/3,1/3), \quad {\varvec{A}}^{\texttt {piso}} = \text {diag}(1/2,1/2,0) \nonumber \\  &   \text {as well as} \quad {\varvec{A}}^{\texttt {ud}} = \text {diag}(1,0,0), \end{aligned}$$

the elastic parameters of PA 6.6 (see Table 2) and a fiber-volume fraction of $19.3\%$, the statistical data for ten runs is summarized in Table 3, where we report on the orthotropic engineering constants.

We observe that for each individual modulus, the standard deviation decreases upon increasing unit-cell length $Q$. The highest observed standard deviation is approximately $0.52\%$ for the planar isotropic case and the shortest edge length $Q=300\,\upmu$m. Moreover, we observe that the systematic error is rather low. More precisely, the unidirectional case shows the highest difference in the Young’s modulus $E_1$ when comparing the largest and the smallest edge lengths. Still, this difference is less than $1.6\%$.

Thus, we conclude that even the small cells may be considered representative for engineering accuracy. We wish to stress that this apparent small size of the representative volume element is a consequence of the combined use of periodized volume elements, i.e., periodic boundary conditions when generating the microstructures, periodic boundary conditions for the displacement field and the high-fidelity realization of the prescribed microstructure characteristics like the fiber-volume fraction and the moments of the prescribed fiber-length distribution.

### The effect of the length-orientation coupling

First, we investigate the distribution of the realized fiber-orientation tensors, considered as functions of the fiber-length, resulting from the length-orientation coupling (2.22)

4.5 $$\begin{aligned} f^{\texttt {MEE}}(\ell ,{\varvec{p}}) = \psi  (\ell )\,\varphi ^\texttt {Bingham}_{\ell \, {\varvec{B}}}({\varvec{p}}) \end{aligned}$$

implied by the maximum-entropy length-orientation closure (2.15). We will use the PBT system, see Table 1, for these investigations. In particular, we will focus on the lognormal distribution for the fiber length, see Fig. 4a for an illustration.

We prescribe the fiber-length distribution together with the second-order fiber-orientation tensor, and obtain the corresponding Bingham parameter by the procedure described in Sect. 3.3. In the process of solving Eq. (3.16), a number of integration weights and integration points are chosen based on the specified fiber-length distribution $\psi$. To evaluate the integral (3.16), the second-order fiber-orientation tensors at these selected integration points, i.e., the individual lengths, need to be computed anyway.

**Fig. 5:**
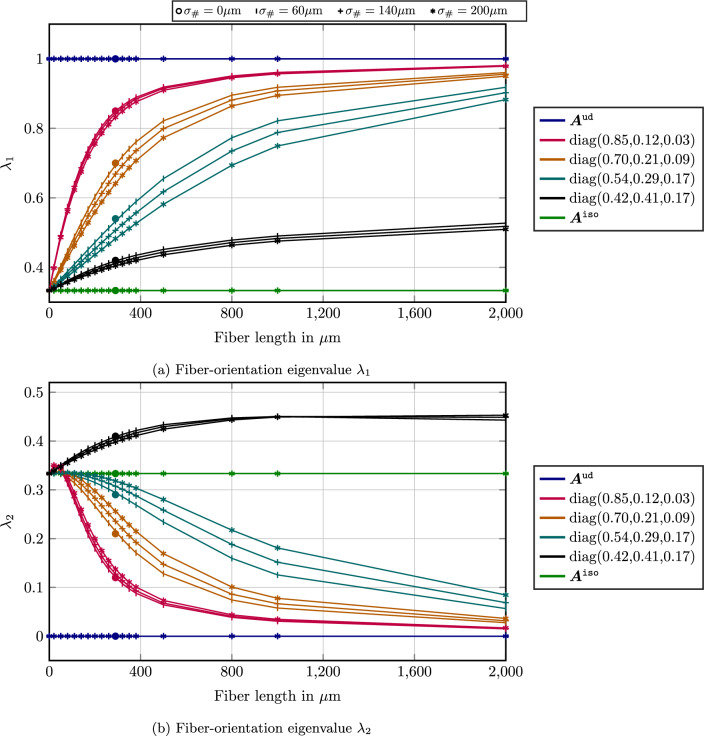
Effect on orientation length coupling for fixed mean length $\mu _{\#} = 290 \,\upmu$m and different standard deviations $\sigma _{\#}$ for different prescribed FOTs

We will use these integration points to “probe” the fiber length-orientation distribution as follows.

We prescribe a number of second-order fiber-orientation tensors whose components are non-zero only on the diagonal with respect to the previously selected Cartesian coordinate system. As a result of the Bingham closure (2.19), the identified Bingham parameter ${\varvec{B}}$ will be diagonal, as well. As a consequence, the computed fiber-orientation tensors will be diagonal. Following the classical convention [19], we will denote these diagonal elements by $\lambda _1$, $\lambda _2$ and $\lambda _3$. Moreover, we will assume that these eigenvalues are ordered in a non-increasing way, i.e., the inequalities

4.6 $$\begin{aligned} \lambda _1 \ge \lambda _2 \ge \lambda _3 \end{aligned}$$

hold. As the second-order fiber-orientation tensor is positive semidefinite and has trace unity, the eigenvalues $\lambda _i$ $(i=1,2,3)$ are non-negative and sum to unity.

With these explanations at hand, we refer to Fig. 5 for the effects of the length-orientation coupling on the realized second-order fiber-orientation tensors. We considered a fixed (number-weighted) mean fiber length $\mu _{\#} =290 \,\upmu$m and different standard deviations $\sigma _{\#}$. The case of vanishing variance is indicated by bullets. We show the two largest eigenvalues $\lambda _1$ and $\lambda _2$ only, as the third eigenvalue may be recovered easily via the trace constraint, i.e.,

4.7 $$\begin{aligned} \lambda _3 = 1 - \lambda _1 - \lambda _2. \end{aligned}$$

Figure 5 reveals that there is no dependence of the fiber orientation on the length for both the isotropic and the unidirectional, i.e., aligned, fiber-orientation states. To understand this effect, we take a look at the maximum-entropy closure (2.10)

4.8 $$\begin{aligned} \varphi ^\texttt {Bingham}_{{\varvec{M}}}({\varvec{p}}) = \exp \left( {\varvec{p}}^T {\varvec{M}}{\varvec{p}}- c({\varvec{M}}) \right) , \quad {\varvec{p}}\in S^2. \end{aligned}$$

The fiber-orientation distribution function $\varphi ^\texttt {Bingham}_{{\varvec{M}}}$ depends on the symmetric $3 \times 3$ matrix ${\varvec{M}}$ and involves the normalization constant (2.11)

4.9 $$\begin{aligned} c({\varvec{M}}) = \log \displaystyle \int _{S^2} { \exp \left( {\varvec{p}}^T {\varvec{M}}{\varvec{p}}\right) } \, dS. \end{aligned}$$

The isotropic fiber orientation corresponds to the Bingham parameter ${\varvec{M}}\equiv 0$. Thus, the rescaling implied by the maximum-entropy method (4.5) has no effect for this orientation state. Similarly, for the planar isotropic and the aligned fiber-orientation states, no coupling effects appear, as these correspond to maximum-entropy closures in two and one dimensions, respectively. In particular, similar arguments as for the (three-dimensional) isotropic fiber-orientation state apply.

Apart from these “pathological” cases, Fig. 5 shows a distinct coupling of length and orientation. We observe that, as the fibers get shorter, the orientation state approaches the isotropic case. Indeed, as mentioned earlier, the isotropic orientation is described by a vanishing Bingham parameter ${\varvec{M}}= 0$. Thus, due to the maximum-entropy coupling (4.5), we automatically obtain a vanishing Bingham parameter ${\varvec{M}}\equiv \ell {\varvec{B}}$ for asmyptotically vanishing fiber length $\ell \rightarrow 0$.

Taking a look at increasing fiber length, we observe a *higher degree of alignment* for longer fibers. Actually, it appears that the orientation state approaches the unidirectional state as $\ell \rightarrow \infty$. However, there are also cases where the planar isotropic fiber orientation is approached as $\ell \rightarrow \infty$.

Figure 5 also shows the influence of the imposed standard deviation. For the largest eigenvalue $\lambda _1$, increasing the standard deviation leads to a decrease. Indeed, due to the higher variance, more “mass” is present for the longer fibers. To ensure that the prescribed fiber-orientation tensor is recovered, this increase needs to be counter-balanced with less strongly oriented fiber states. The opposite trend emerges for the second eigenvalue $\lambda _2$.

To get an insight into the resulting effective mechanical behavior we study the effective stiffness corresponding to the PBT material system, see Table 1, and a second-order fiber-orientation tensor

4.10 $$\begin{aligned} {\varvec{A}}= \text {diag}(0.74,0.23,0.03). \end{aligned}$$

**Fig. 6:**
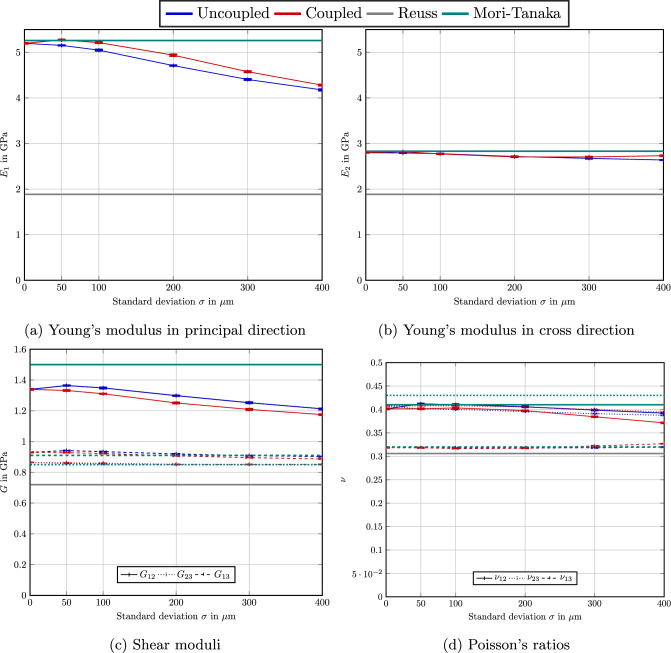
Computed effective engineering constants for the PBT material system, see Table 1, fiber-orientation tensor ${\varvec{A}}=\text {diag}(0.74,0.23,0.03)$ and varying standard deviation for ten different realizations

**Fig. 7:**
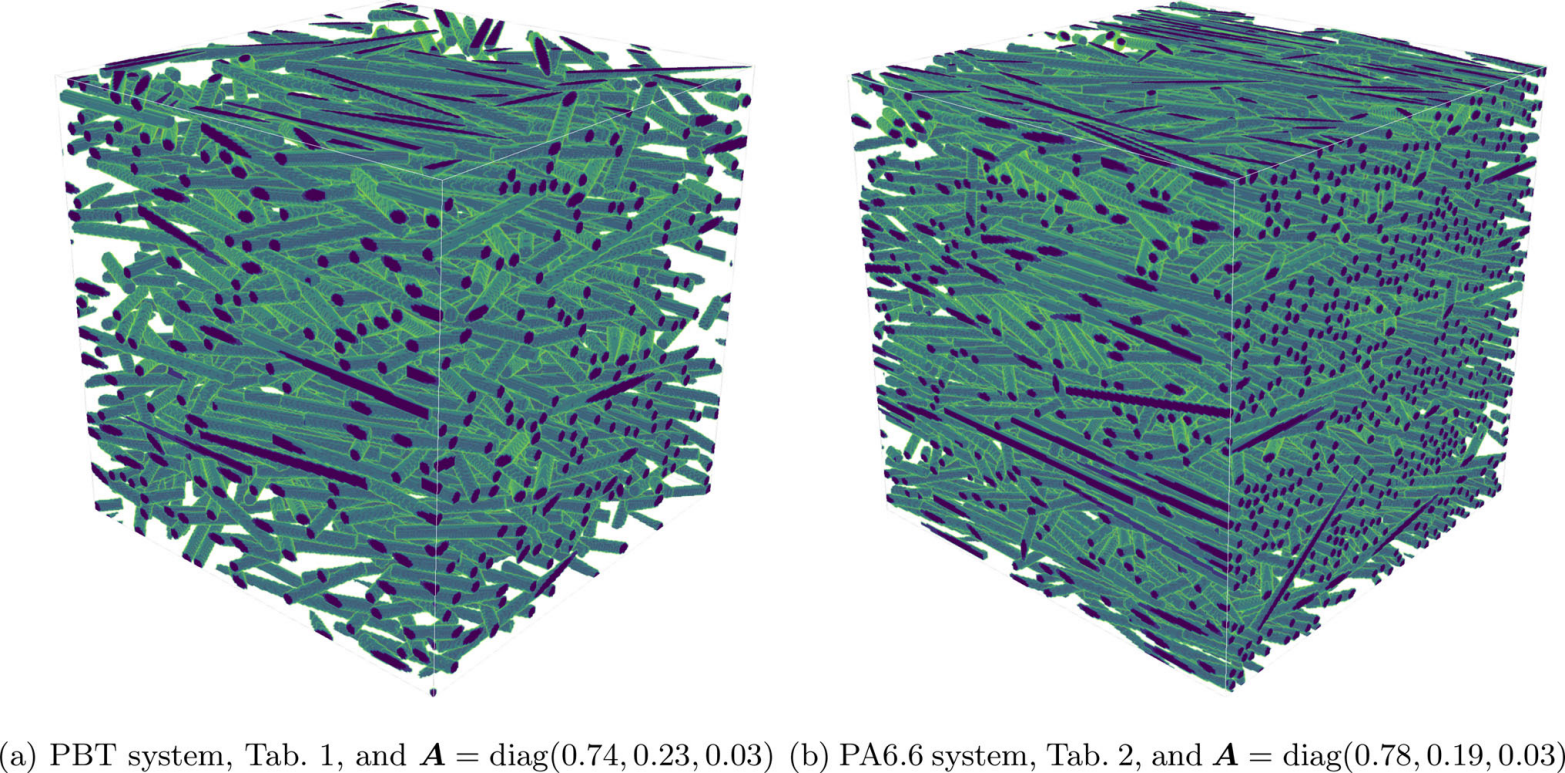
Sample images of a unit cell for the two distinct cases described in Tables 1 and 2

This orientation state features an almost planar fiber orientation with about three quarters of the fibers oriented in principal direction and the remaining quarter approximately pointing in the cross direction. To get an impression into how corresponding unit cells look like, we showed a sample in Fig. 7a. Such a fiber-orientation state is actually quite common for injection-molded short-fiber composites [18, 19].

To assess the fluctuations of the computed results, we considered ten different realizations for each scenario. Figure 6 shows the computed Young’s moduli in 1- and 2-direction, comparing the uncoupled model (2.14) and the coupled model (2.19), i.e., the based on the maximum-entropy assumption. Here, the Young’s moduli $E_1$ and $E_2$ point in the directions of the prescribed fiber-orientation tensor ${\varvec{A}}$ corresponding to the two largest eigenvalues. We also report on the realized orthotropic shear moduli and corresponding Poisson’s ratios. Please note that we use the maximum-entropy closure, i.e., the Bingham distribution, for the uncoupled model, as well. The considered fiber-length distributions for the different standard deviations are shown in Fig. 4a.

We notice that, except for close to vanishing standard deviation, increasing the standard deviation decreases the Young’s modulus $E_1$ consistently. There is a considerable drop between no standard deviation and a standard deviation of $\sigma _{\#} = 400\,\upmu$m, i.e., about one GPa - which corresponds to about $20\%$ of the stiffness. In contrast, the Young’s modulus $E_2$, see Fig. 6b, turns out to be less influence by this change in variance. Moreover, the standard deviations are rather insignificant for all considered moduli.

Comparing the uncoupled and the coupled model, we observe in Fig. 6a that the Young’s modulus $E_1$ increases significantly for the coupled model and non-zero standard deviation. This observation is a direct consequence of the higher alignment of the longer fibers ensured by the maximum-entropy length-orientation closure (2.19). Overall, the difference is no larger than $5\%$, reached at $200\,\upmu$m. This relative difference is actually rather significant. However, due to the comparatively low filler content for the PBT material system, the absolute differences are not that large, i.e., about a quarter GPa. For the cross direction, shown in Fig. 6b, the differences between the coupled and the uncoupled model are rather small. Taking a closer look on the shear moduli and the Poisson’s ratios, we observe that the relative deviations between the coupled and the uncoupled model are on the same order of magnitude as the deviations observed for the Young’s modulus $E_1$.

For the convenience of the reader, we also included selected mean-field estimates for the effective stiffness of the fiber material system into Fig. 6. The first-order estimates comprise the Voigt estimate, i.e., the volume average of the constituent elasticity tensors, and the Reuss estimate, which arises from a volume average of the constituents’ compliance tensors. The Voigt estimates

4.11 $$\begin{aligned} E^{\text {Voigt}} = 10.94\text {GPa} \quad \text {and} \quad G^{\text {Voigt}}=4.43\text {GPa} \end{aligned}$$

for the effective Young’s modulus and the effective shear modulus turn out to be quite high, actually. As these values are outside the limits of Fig. 6, we omitted them from the presentation. The Reuss bounds

4.12 $$\begin{aligned} E^{\text {Reuss}} = 1.89\text {GPa} \quad \text {and} \quad G^{\text {Reuss}}=0.72\text {GPa} \end{aligned}$$

turn out to be closer to the computational results, and are included in Fig. 6. Still, the Reuss predictions for the directional Young’s moduli lead to an underestimation by about 1/3, at least.

We also included the Reuss estimate for Poisson’s ratio in Fig. 6d. The Voigt and Reuss estimates for Poisson’s ratio actually correspond to Poisson’s ratio of the Voigt/Reuss average of the shear and compression moduli of the phases, i.e., they *do not* arise from an average of the Poisson’s ratios of the individual phases. Thus, some care has to be taken. Higher fidelity is reached when using Hashin-Shtrikman bounds, for instance, see Stefaunik et al. [94].

In addition to the first-order estimates, mean-field models with higher fidelity may be used. For the case at hand, we report on the (orientation-averaged) Mori–Tanaka estimate [76, 95], which coincide with the celebrated lower Hashin-Shtrikman bounds under specific circumstances [2, 3].

For the material system at hand, the Mori–Tanaka model provides a good agreement with the computational results in case of the directional Young’s moduli and Poisson’s ratio in case of vanishing standard deviation $\sigma$. The out-of-plane shear modulus is severely overestimated, though. We used a Mori–Tanaka model that is insensitive to the details of the length-orientation distribution, but takes into account the total fiber-orientation tensor of second order, only, together with the fiber-orientation closure approximation used. Extending the Mori–Tanaka estimate to length-orientation coupling is a possible direction of further research.

These findings should be contrasted with the investigations made for the second material system, shown in Table 2, at hand. This composite comes with a significantly higher filler content and a slightly larger aspect ratio. However, the differences between the elastic parameters of fibers and matrix are smaller. Notice that the *Weibull* distribution [96] was shown to reproduce the length distribution of this composite better [45, 76, 95]. This fact contrasts with the PBT system where the *lognormal* distribution provides a significantly better match with experimental data.

**Fig. 8:**
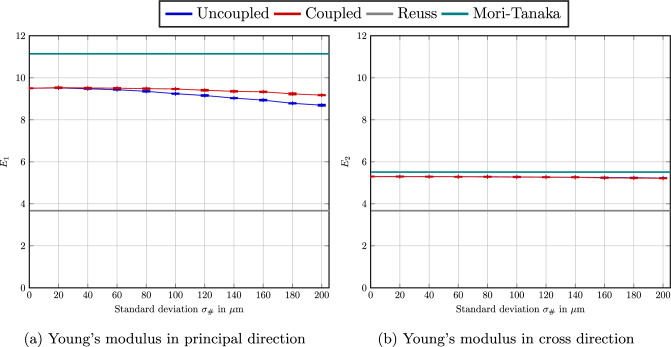
Computed effective Young’s moduli for the polyamide material system, see Table 2, fiber orientation ${\varvec{A}}=\text {diag}(0.78,0.19,0.03)$ and varying standard deviation for 10 different realizations

The resulting Young’s moduli are shown in Fig. 8 for the length distributions shown in Fig. 4b and the prescribed fiber-orientation tensor

4.13 $$\begin{aligned} {\varvec{A}}=\text {diag}(0.78,0.19,0.03) \end{aligned}$$

of second order. A sample microstructure is shown in Fig. 7b. We restrict to showing the Young’s moduli in 1- and 2-directions, as these scenarios represented the extreme cases for the material system we previously examined. We make similar observations as for the PBT case - the coupled model predicts significantly stiffer results compared to the uncoupled model for comparatively large standard deviations, at least for the Young’s modulus $E_1$. The influence on the transverse modulus $E_2$ is small, as only the third significant digit is affected.

However, due to the increased filler content, the absolute differences in the computed Young’s moduli are much larger. At a standard deviation of $\sigma _{\#} = 200 \,\upmu$m, the relative difference of about $5\%$ between the coupled and the uncoupled model translates into about half a GPa.

We also report on the first-order analytical estimates, the Voigt and Reuss bounds

4.14 $$\begin{aligned} E^{\text {Voigt}} = 16.65\text {GPa} \quad \text {and} \quad E^{\text {Reuss}} = 3.66\text {GPa} \end{aligned}$$

for Young’s modulus. We observe that the Voigt bound significantly overestimates the appearing Young’s modulus. The Reuss estimate is closer to the computed results, but underestimates the Young’s modulus in principal direction by a factor of more than two.

For the convenience of the reader, we also include the (orientation-averaged) Mori–Tanaka model [2, 3] into Fig. 8. We observe that this analytical model overestimates the Young’s modulus in principal direction by more than $10\%$. This inferior accuracy compared to the PBT material system may be a consequence of the significantly higher filler content, leading to higher stresses in the matrix, in particular in regions where fibers are close to each other. Such a situation is not directly included in the (conventional) Mori–Tanaka estimates.

### Comparison to experimental data

This section is devoted to applying the developed methodology to industrial examples and comparing the ensuing results. We will both consider the glass-fiber reinforced PBT material system and the glass-fiber reinforced polyamide which we considered previously.

**Table 4:** Statistical data for the three layers of the PBT-GF composite [8] shown in Fig. 1

Layer	$\mu _{\#}$	$\mu _{\ell }$	$\sigma _{\#}$	${\varvec{A}}$
	in $\upmu$m	in $\upmu$m	in $\upmu$m	
Skin	228.78	543.58	268.42	$\text {diag}(0.74,0.23,0.03)$
Transition	207.30	478.34	183.32	$\text {diag}(0.54,0.41,0.05)$
Core	235.00	563.01	277.13	$\text {diag}(0.21,0.75,0.04)$

We will treat the PBT composite first, drawing from the experimental data of Müller [3]. Recall that at least three different layers could be distinguished in the micro-computed computer tomography scan, see Fig. 1, corresponding to the skin as well as the core layer with an additional transition layer in between. In a first step, the discrete fiber-length data was used to identify the parameters of a lognormal distribution by classical regression with a quadratic goodness of fit objective function.

**Fig. 9:**
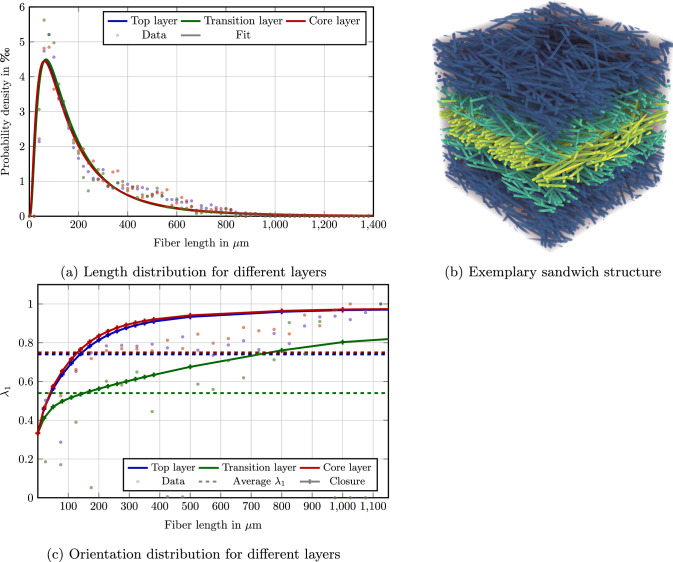
Length and orientation distribution of layered microstructure

For each of the layers, the identified (number-weighted) mean $\mu _{\#}$ and standard deviation are shown in Table 4 and illustrated in Fig. 9a. We also have mentioned volume-weighted mean $\mu _{\ell }$ for reference. We observe that the three identified lognormal distributions do not differ significantly and match the fiber-length data with reasonable accuracy. The experimental data reports a peak at about $100 \,\upmu$m which is not covered by the length distribution. This effect could be, however, a result of the fiber-segmentation procedure. Moreover, the fiber-length distribution appears to underestimate the fiber length in the region between $400\,\upmu$m and $800\,\upmu$m. However, we wish to remark a couple of things. For a start, the Weibull distribution leads to a much worse fit. Secondly, an identification based on the statistical moments of the measured fiber-length data also leads to a much worse fit, essentially due to the non-equispaced distribution of the fiber-length data. Last but not least, it appears apparent that a more sophisticated fiber-length distribution, e.g., based on a mixture of models, could improve the fit, but may impoverish the simplicity of the approach at hand.

We used the layer-wise identified second-order fiber-orientation tensors recorded in Table 4 to compute the maximum-entropy length-orientation coupling (2.19). The results are shown in Fig. 9c in the following form. The largest eigenvalue $\lambda _1$ is shown as a function of the fiber length. Moreover, the largest eigenvalue of the *average* second-order fiber-orientation tensor is shown with a dashed line, as well. The experimental data is indicated by thickened dots, corresponding to each of the three layers at hand.

As the ordered eigenvalues of the second-order fiber-orientations in both the skin and the core layers were similar, together with the fiber-length distributions, the estimated identified fiber length-orientation distributions are similar, as well. This similarity is seen in the data, as well. In contrast, the data for the transition layers shows a much weaker alignment, and this tendency is reflected by the proposed closure approximation, as well. We observe that the tendency of the fibers to align more strongly at higher lengths is reflected by the closed distribution, as well. Concerning the qualitative agreement, we observe a rather good match for the transition layer, whereas the alignment of to top and the core layers is overestimated by the proposed model for the shorter fibers with lengths up to $800\,\upmu$m.

With the identified fiber length-orientation distributions we generated five-layered (skin-transition-core-transition-skin) sandwich microstructures, e.g., shown in Fig. 9b. We use a similar coloring of the layers as for the $\upmu$CT scan shown in Fig. 1 to enable a quick qualitative evaluation of the generated microstructure. We generated this type of microstructures for both the case coupled (2.19) and uncoupled (2.14) fiber length-orientation and compare the resulting engineering constants with the mechanical experiments in Table 5. The Experimental data is taken from the work of Müller  [3]. For both the coupled and the uncoupled case, five different realization were evaluated and averaged. $E_1$ and $E_2$ represents the Young’s moduli in the ${\varvec{e}}_1$ (longitudinal) and the ${\varvec{e}}_2$ (transverse) direction. From the table we observe that the coupled model predicts the longitudinal Young’s modulus ($E_1$) more accurately than its uncoupled counterpart. On the other hand, the Young’s modulus in transverse direction ($E_2$) is slightly over-estimated. However, both the coupled and the un-coupled model lead to predictions lying within the standard deviations of the measured effective properties.

**Table 5:** Runtimes and computed Young’s moduli versus experiments [3] for the PBT-GF composite

	Experiments	Sandwich	Sandwich
	[3, Fig. 3.1]	(uncoupled)	(coupled)
$E_1$ in GPa	$4.48\pm 0.13$	$4.38\pm 0.02$	$4.51\pm 0.02$
$E_2$ in GPa	$3.45\pm 0.07$	$3.45\pm 0.01$	$3.50\pm 0.01$
runtime in s	–	$97.22\pm 25.92$	$75.59\pm 21.95$

To further validate the proposed model, we consider the polyamide material system and the associated strongly different microstructure. The Material properties and other relevant information are recorded in Table 2. In previous work [45], it was shown that the fiber-length data could be rather accurately described by the Weibull distribution. Moreover, only two layers could be clearly distinguished on the $\upmu$CT scan. Thus, a three-layers sandwich microstructure (skin-core-skin) is considered in the computational model. For more information about the Weibull length distribution and the considered microstructure, we refer to our previous paper [45]. Experimental data is taken from the work of Hessman et al.  [76, 95]. The previous material system showed that the different layers show a rather similar length distribution. We used this characteristic for the system at hand, as well, and considered the same length distribution for both layers. Information about different layers is presented in Table 6.

**Table 6:** Statistical data for the layers of the PA-GF composite studied by Hessman et al. [76, 95]

Layer	$\mu _{\#}$	$\mu _{\ell }$	$\sigma _{\#}$	${\varvec{A}}$
	in $\upmu$m	in $\upmu$m	in $\upmu$m	
Skin	273.12	332.65	127.50	$\text {diag}(0.86, 0.12, 0.02)$
Core	273.12	332.65	127.50	$\text {diag}(0.23, 0.74, 0.03)$

**Table 7:** Runtimes and computed Young’s moduli versus experiments [76, 95] for the PA-GF composite

	Experiments	Sandwich	Sandwich
	[76, Fig. 2]	(uncoupled)	(coupled)
$E_1$ in GPa	$10.34\pm 0.4$	$10.42\pm 0.03$	$10.10\pm 0.01$
$E_2$ in GPa	$5.50\pm 0.1$	$5.78\pm 0.00$	$5.54\pm 0.00$
Runtime in s	-	$69.00\pm 8.45$	$91.97\pm 12.5$

Following similar steps as for the previous PBT-based system, we are led to computational results shown in Table 7. We observe that the longitudinal Young’s modulus $E_1$ is slightly overestimated for the uncoupled model, but within the experimental variance. In contrast, the transverse Young’s modulus $E_2$ is significantly overestimated, turning out to exceed the confidence interval significantly. In contrast, the coupled model matches the transverse Young’s modulus with high accuracy. However, the longitudinal Young’s modulus is slightly lower. All in all, we observe a smaller difference between the longitudinal and the transverse Young’s modulus for the coupled model compared to the uncoupled model. In particular, the differences of the experimental results are matched more accurately for the material system at hand using the coupled model instead of the uncoupled model.

## Conclusion

This work was devoted to establishing a closure approximation for the full fiber length-orientation distribution function for prescribed fiber-length distribution and second-order fiber-orientation tensor. We proposed an information-theoretic approach from first principles based on the well-known maximization of the information-theoretic entropy. The resulting distribution turned out to be a parametrized version of the Bingham distribution, involving a non-trivial coupling of the fiber length and the fiber orientation. We discussed a robust and efficient numerical strategy for identifying the necessary model parameters. Once these are identified, the maximum-entropy model automatically predicted that long fibers tend to align more than shorter fibers do, which is in agreement with experimental findings.

We wish to highlight the flexibility of our strategy with respect to the fiber-length distribution - both discrete and continuous distributions may be used. We demonstrated the ease of incorporating different (continuous) fiber-length distributions.

We integrated the proposed length-orientation model into the sequential-addition and migration algorithm [42] based on prescribing the closed length-averaged fiber-orientation tensor [45, 46]. Using such a microstructure characteristic led to a rather small variance of the computed effective elastic properties, reinforcing the confidence in this assumption, which might be of interest for microstructure reconstruction [97–99].

When comparing the results to experiments, we could show that the proposed coupled model matches experimentally measured Young’s moduli with higher accuracy than for the uncoupled model, at least if a least-squares fit of the fiber-length distribution based on measured data is considered.

The authors are not aware of a similar length-orientation closure in the literature [100], and investigating a greater variety of models, in particular extending existing orientation closures, would be rather interesting. Moreover, covering a wider class of material systems with fiber-like inclusions, e.g., carbon nano-tubes [101] may be of interest, as well. Last but not least, an integration into dedicated multi-scale schemes [43, 44, 102] appears desirable.

## A FFT-based computational micromechanics in a nutshell

The paragraph at hand seeks to provide an idea of the inner workings of FFT-based computational micromechanics methods [77, 78], whose computational efficiency led to a variety of extensions and applications in the last decades [84, 103, 104].

We are concerned with a unit cell $Y=[0,Q_1] \times [0,Q_2] \times [0,Q_3]$ in three spatial dimensions and suppose that a stiffness distribution $\mathbb {C}$ is given on the cell *Y*. More precisely, the stiffness distribution associates a stiffness tensor to each microscopic point ${\varvec{x}}\in Y$ in the unit cell. For the fiber-matrix composites considered in the article at hand, all continuum points within the fiber are associated with the stiffness $\mathbb {C}^{\text {f}}$ of the fiber, whereas the matrix material is encoded by another stiffness tensor $\mathbb {C}^{\text {m}}$.

For a prescribed macroscopic strain tensor $\overline{\varvec{\varepsilon }}$, periodic homogenization seeks the periodic displacement fluctuation field ${\varvec{u}}_{\overline{\varvec{\varepsilon }}}:Y \rightarrow \mathbb {R}^3$ which solves the balance equation

6.1 $$\begin{aligned} \text {div }\mathbb {C}:(\overline{\varvec{\varepsilon }} + \nabla ^s {\varvec{u}}_{\overline{\varvec{\varepsilon }}}) = 0 \end{aligned}$$

on the unit cell *Y*, where neither inertial effects nor microscopic body forces are considered and we restrict the discussion to small strains.

Once the equation (6.1) is solved, the corresponding apparent stress is defined by a volume average of the local stress

6.2 $$\begin{aligned} \varvec{\sigma }_{\overline{\varvec{\varepsilon }}} = \frac{1}{Q_1 Q_2 Q_3} \int _Y \mathbb {C}:(\overline{\varvec{\varepsilon }} + \nabla ^s {\varvec{u}}_{\overline{\varvec{\varepsilon }}}) \, d{\varvec{x}}. \end{aligned}$$

Subsequently, the solutions ${\varvec{u}}_{\overline{\varvec{\varepsilon }}}$ corresponding to *different* imposed average strains are combined to form the apparent stiffness $\mathbb {C}^{\texttt {app}}$, implicitly determined from the identity

6.3 $$\begin{aligned} \mathbb {C}^{\texttt {app}} : \overline{\varvec{\varepsilon }} = \varvec{\sigma }_{\overline{\varvec{\varepsilon }}}. \end{aligned}$$

Thus, the essential problem is to resolve the equilibrium equation (6.1). Classically, this is achieved by bringing the balance equation (6.1) into *weak* form

6.4 $$\begin{aligned} \int _Y \nabla ^s {\varvec{v}}: \mathbb {C}:(\overline{\varvec{\varepsilon }} + \nabla ^s {\varvec{u}}_{\overline{\varvec{\varepsilon }}})\, d{\varvec{x}}= 0 \quad \text {for all} \quad {\varvec{v}}\in H^1_{\#}(Y;\mathbb {R}^3), \end{aligned}$$

where $H^1_{\#}(Y;\mathbb {R}^3)$ denotes the first-order Sobolev space of periodic vector fields. Then, a finite-element discretization arises by choosing a specific finite-dimensional subspace $V_h \subseteq H^1_{\#}(Y;\mathbb {R}^3)$, where *h* is a mesh-related parameter, and to seek the solution ${\varvec{u}}_{\overline{\varvec{\varepsilon }},h} \in V_h$ which solves the equation

6.5 $$\begin{aligned} \int _Y \nabla ^s {\varvec{v}}_h: \mathbb {C}:(\overline{\varvec{\varepsilon }} + \nabla ^s {\varvec{u}}_{\overline{\varvec{\varepsilon }},h})\, d{\varvec{x}}= 0 \quad \text {for all} \quad {\varvec{v}}_h \in V_h. \end{aligned}$$

Unfortunately, due to the inherent complexity of industrial-scale microstructures, the resulting linear system

6.6 $$\begin{aligned} \underline{\underline{A}}_h \, \underline{u}_h = \underline{f}_h \end{aligned}$$

turns out to be rather huge. In particular, the classical strategy of assembling the finite-element stiffness matrix $\underline{\underline{A}}_h$ requires a significant chunk of memory, requiring high-performance computing facilities to be used. To reduce the memory footprint, matrix-free solution strategies may be utilized. These strategies, however, require iterative solvers like the conjugate-gradient method to be put into service. The iteration count of such iterative solvers depends on the condition number of the finite-element stiffness matrix $\underline{\underline{A}}_h$. Unfortunately, the iteration count of (optimal) solvers scales inversely proportional to the mesh size *h*. Thus, a finer mesh also requires more iterations - in addition to having to handle more degrees of freedom. As a remedy, suitable (matrix-free) preconditioning strategies may be used to cure the mesh-spacing induced ill-conditioning of the finite-element stiffness matrix, i.e., instead of the linear system (6.6), the *equivalent* linear problem

6.7 $$\begin{aligned} \underline{\underline{P}}_h\,\underline{\underline{A}}_h \, \underline{u}_h = \underline{\underline{P}}_h\,\underline{f}_h \end{aligned}$$

is solved, where $\underline{\underline{P}}_h$ is an invertible matrix, called preconditioner. The essential insight is that the iteration count to solve the preconditioned system (6.7) depends on the condition number of the matrix $\underline{\underline{P}}_h\underline{\underline{A}}_h$ (instead of the condition number of the matrix $\underline{\underline{A}}_h$ for the original problem (6.6)). Thus, by appropriately choosing the preconditioner $\underline{\underline{P}}_h$, the iteration count for solving the preconditioned problem (6.7) may be significantly less than for solving the original problem (6.6). However, the challenge is to find a preconditioner $\underline{\underline{P}}_h$ which is also inexpensive to compute.

Methods based on the fast Fourier transform (FFT) are based on the following idea. If a discretization on a regular, i.e., Cartesian, grid is used and periodic boundary conditions are employed, then, for every homogeneous stiffness tensor $\mathbb {C}^0$ and any field ${\varvec{f}}_h \in V_h$, the finite-element problem

6.8 $$\begin{aligned} \int _Y \nabla ^s {\varvec{v}}_h : \mathbb {C}^0 : \nabla ^s {\varvec{u}}_h \, d{\varvec{x}}= \int _Y {\varvec{v}}_h \cdot {\varvec{f}}_h \, d{\varvec{x}}\quad \text {for all} \quad {\varvec{v}}_h \in V_h \end{aligned}$$

may be solved explicitly using the FFT. The approach, which works *for any* finite-element discretization, as long as it employs a regular grid, is based on making an ansatz of the involved fields ${\varvec{u}}_h$, ${\varvec{v}}_h$ and ${\varvec{f}}_h$ by suitable (truncated) Fourier series and working out the corresponding coefficients in Fourier space. We refer to the dedicated works [105–107] for further details.

Writing down the auxiliary problem (6.8) in matrix–vector form

6.9 $$\begin{aligned} \underline{\underline{A}}_h^0 \, \underline{u}_h = \underline{f}_h, \end{aligned}$$

the FFT may thus be used to compute the inverse of the matrix $\underline{\underline{A}}_h^0$. This inverse is then used as a preconditioner

6.10 $$\begin{aligned} \underline{\underline{P}}_h = \left( \underline{\underline{A}}_h^0 \right) ^{-1} \end{aligned}$$

for the original problem (6.6). The resulting computational strategy (6.7) comprises solvers whose iteration count is bounded independently of the mesh spacing and which essentially operate in place, i.e., come with a comparatively low memory footprint.

There is actually an entire zoo of FFT-based methods, which also comprise finite-difference discretizations [80, 108, 109] and spectral discretizations [110–112]. The different discretizations schemes were studied and compared in great detail, see, for instance the work [113] on hourglass control, or the review article [84].

Also, the equivalent so-called Lippmann–Schwinger form

6.11 $$\begin{aligned} \underline{u}_h = \left( \underline{\underline{A}}_h^0 \right) ^{-1}\,\left[ \underline{f}_h - (\underline{\underline{A}}_h-\underline{\underline{A}}_h^0) \, \underline{u}_h \right] \end{aligned}$$

of the Eq. (6.7) for the choice (6.10) is rather popular and serves as a frequent starting point of implementations.

For the work at hand, we use the discretization on a staggered grid [80], which may be interpreted as a finite element discretization with a special quadrature rule, as it combines artifact-free high-quality solution fields and a low computational overhead.

## Footnotes

1 Angular central Gaussian distribution [63].

2 $$\det {\varvec{M}}= 1$$

3 Bingham distribution [64].
